# Integrative single-cell RNA and ATAC sequencing reveals that the FOXO1-PRDX2-TNF axis regulates tendinopathy

**DOI:** 10.3389/fimmu.2023.1092778

**Published:** 2023-05-08

**Authors:** Junfeng Guo, Hong Tang, Pan Huang, Xiao Ye, Chuyue Tang, Zhao Shu, Junfeng Guo, Xia Kang, Youxing Shi, Binghua Zhou, Taotao Liang, Kanglai Tang

**Affiliations:** ^1^ Department of Orthopedics/Sports Medicine Center, State Key Laboratory of Trauma, Burn and Combined Injury, Southwest Hospital, Third Military Medical University, Chongqing, China; ^2^ Department of Gastroenterology, The First Affiliated Hospital of Chongqing Medical University, Chongqing, China; ^3^ Department of Stomatology, The 970th Hospital of the Joint Logistics Support Force, Yantai, China

**Keywords:** tendinopathy, single-cell multi-modal analysis, microenvironment, harmful cells, Prdx2

## Abstract

**Introduction:**

Tendinopathy, the most common form of chronic tendon disorder, leads to persistent tendon pain and loss of function. Profiling the heterogeneous cellular composition in the tendon microenvironment helps to elucidate rational molecular mechanisms of tendinopathy.

**Methods and results:**

In this study, through a multi-modal analysis, a single-cell RNA- and ATAC-seq integrated tendinopathy landscape was generated for the first time. We found that a specific cell subpopulation with low *PRDX2* expression exhibited a higher level of inflammation, lower proliferation and migration ability, which not only promoted tendon injury but also led to microenvironment deterioration. Mechanistically, a motif enrichment analysis of chromatin accessibility showed that *FOXO1* was an upstream regulator of PRDX2 transcription, and we confirmed that functional blockade of *FOXO1* activity induced *PRDX2* silencing. The TNF signaling pathway was significantly activated in the *PRDX2*-low group, and TNF inhibition effectively restored diseased cell degradation.

**Discussion:**

We revealed an essential role of diseased cells in tendinopathy and proposed the FOXO1-PRDX2-TNF axis is a potential regulatory mechanism for the treatment of tendinopathy.

## Introduction

Tendons, which transmit contractile force, are crucial components of the musculoskeletal system. Tendinopathy is the most common form of chronic tendon disorder, accounting for 30% of musculoskeletal counseling in general practice (1). The main clinical manifestations are pain, a decline in function and reduced exercise tolerance (2). Histological analyses often reveal tissue degeneration and disorganization, predisposing the tendon to eventual tearing and rupture (3). Tendinopathy imposes a considerable burden on individuals and society due to slow and frequently poor tendon healing (4). Thus, the repair of tendon injuries is a major clinical challenge in orthopedic medicine (5).

Although the risk factors for tendinopathy, such as overuse or age-related degeneration, are relatively clear, the pathogenesis of tendinopathy remains unclear (6). In recent years, the heterogeneity of disease has gradually become a focus of attention and has been detected in many biological tissues and disease states related to treatment and progression (7). Tendinopathy is markedly heterogeneous, and recent discoveries suggest that tendon tissue includes many different cell subsets with distinct functions that influence the occurrence, progression and healing of tendinopathy (8, 9). Thus, elucidating the heterogeneous cell composition of tendons hold great promise for the treatments for tendinopathy.

Single-cell sequencing provides a cutting-edge technique to capture the marked heterogeneity of tendinopathy, characterize the complex tendon microenvironment and identify new cell types and states (10). Assay for transposase-accessible chromatin (ATAC) sequencing (ATAC-seq) reveals an epigenomic landscape and rationale for mammalian DNA regulatory variation by identifying distinct patterns of chromatin accessibility (11). As technology has advanced, the latest multi-modal ATAC and gene expression analyses enable simultaneous profiling of the transcriptome (using RNA-seq) and epigenome (using ATAC-seq) of a single cell, generating a unified view of the gene expression profile and epigenomic landscape. By leveraging the two modalities at once, the analysis can be performed to identify drivers of differential gene expression and identify cells with similar transcriptional profiles but functionally different chromatin landscapes. These efforts show relevance for exploring drivers of tendinopathy heterogeneity and contributing to our understanding of gene expression and regulation in different cell types.

In this study, we first drew a single-cell RNA and ATAC integrative tendinopathy landscape, allowing us to explore the tendon microenvironment through multi-modal analysis. We elucidated the important role played by a specific tendon-derived stem cells (TDSCs) subpopulation in promoting tendon injury and identified the complicated regulatory relationships, which may help to precise treatment of tendinopathy.

## Materials and methods

### Human sample pre-treatment

The specimens of tendinopathy (N=3) were procured from male adults between the ages of 20 and 35, who were diagnosed with tendinopathy through medical records and MRI scans. Following the surgical procedure, the diagnosis was authenticated *via* histopathological examination. Individuals who are undergoing long-term hormone therapy will not be considered. The peritendinous connective tissues were completely removed from the harvested tendons before processing. After the tissues were digested by collagenase, cell suspension was washed with the medium and passed through a cell strainer. Cells were seeded and incubated at 37°C/5% CO2 for 14 days. Passage 2 cells were used for further study. TDSCs were validated by fluorescence activated cell sorting with specific cell surface markers for mesenchymal stem cells. TDSCs was collected in a 1.5-ml microcentrifuge tube, 300 µl NP40 lysis buffer was added, and the mixture was incubated on ice for 5 min. Then, the suspension was filtered through a 70-µm filter and transferred to a new 2-ml microcentrifuge tube. After gradient centrifugation at 500 rcf for 5 min at 4°C, the supernatant was removed. 1ml PBS, 1% bovine serum albumin (BSA), and an RNase inhibitor were added and incubated on ice for 5 min. Repeated centrifugation at 500 rcf for 5 min at 4°C and removed the supernatant. Resuspend with 1ml PBS + 1% BSA + 1U/µl RNase Inhibitor. Add 10 ul 7AAD ready-made solution to 1-ml sample and incubate for 5 min on ice.

### Multiome library construction and sequencing

Chromium Next GEM Single Cell Multiome ATAC and Gene Expression assay produces two library types from the same single nuclei: the ATAC library and the gene expression library. The obtained nuclear suspension was incubated in a transposase mixture that entered the nuclei and preferentially fragmented DNA in open chromatin regions. Oligonucleotides containing an Illumina P5 sequence, a 16-nt 10x barcode and a spacer sequence were then released. Barcoded, full-length preamplified cDNA was further amplified *via* PCR to generate sufficient mass for gene expression library construction. Through a scalable multi-modal approach, we simultaneously profiled the epigenomic landscape and gene expression in single nuclei. More detailed information is available at https://www.10xgenomics.com/products/single-cell-multiome-atac-plus-gene-expression.

### Data pre-processing

Raw data of ordinary scRNA-seq was downloaded from the GEO database (GSE150482). The gene expression data were processed with the Seurat package, and the ATAC-seq data were processed by the Signac package in R software (V4.0.2). We removed low-quality cells, identified as having more than 10% mitochondrial genes or fewer than 300 feature genes. The number of feature genes in each cell was divided by the total number of genes in the cell and then multiplied by 10000. A total of 2000 highly abundant variable feature genes were identified and used to eliminate batch effects. FindIntegrationAnchors algorithm was used to find a set of anchors between normal and tendinopathy objects. Fifty dimensions with the most significant amount of information were use in the anchor weighting procedure. The variables of “nCount_RNA” and “percent.mito” were regressed out during scaling and centering features in the dataset.

### Isolation and culture of rat TDSCs

The TDSCs used in this study were primary cells isolated and cultured from rats. The harvested tendons underwent a meticulous removal of their peritendinous connective tissues before being processed. Separated Achilles tendon tissue was minced with scissors, digested with type I collagenase (Solarbio, Beijing, China) for 30 min, and centrifuged at 1500 rpm for 5 min. The supernatant was removed and resuspended to obtain a cell suspension. The TDSCs were cultured in low-glucose DMEM (HyClone, South Logan, UT, USA) supplemented with 10% fetal bovine serum (FBS; Pansera ES, Adenbach, Germany) and 1% penicillin–streptomycin (Gibco, Grand Island, NY, USA) at 37°C with 5% CO2. The cells were confirmed with specific cell surface markers for mesenchymal stem cells by fluorescence activated cell sorting.

### Cell cycle analysis

Cell cycle analysis was performed to identify the cell cycle state of distinct clusters on the basis of G1/S- and G2/M-phase-specific genes expression. G1/S and G2/M phase feature genes were identified following procedures described in a previous study (12). We calculated the cycle score for each cell using the CellCycleScoring function in Seurat, the “ctrl” was set to NULL and the “set.ident” was set to FALSE. We identified cells in the G1, S, or G2/M phase based on the scores of these two programs. *MKI67* expression was assessed to validate the cell cycle scores.

### Mechanical stimulation evaluation

Retrieve a collection of genes designated as “response to physical stimuli” from the Gene Ontology database (GO:0009612). The mechanical stimulation level of TDSCs was evaluated through the utilization of the AddModuleScore function available in the Seurat package. The features parameter is comprised of the gene set mentioned above, and the number of control features selected from the same container for each analyzed feature is set to 100. The expression of Piezo1 was assessed to validate the mechanical stimulation level.

### Three-way differentiation capacity evaluation

The feature genes responsible for osteogenic, chondrogenic, and adipogenic differentiation were determined by extracting information from the Msigdb database (No. M14199, M13053 and M8364). The Seurat package’s AddModuleScore function was employed to assess the three-way differentiation potential of TDSCs. The osteogenic, chondrogenic, and adipogenic abilities levels of TDSCs were evaluated by substituting the three gene sets into the feature parameters, respectively.

### Pseudotemporal ordering of TDSCs

Monocle (v2.18.0) orders individual cells *via* differentiation assessment. The reduceDimension function reveals the trajectories of differentiating cells and is used to perform discriminative dimensionality reduction *via* a learning tree (DDRTree). The orderCells function was used to calculate where each cell falls within that trajectory. The BEAM algorithm was used to identify branch variable genes. Genes with significantly variable expression and a q-value less than 10-4 were considered “branch-dependent” genes. GO Enrichment Analysis of these genes was performed using the enrichGO function in clusterProfiler package, the keyType was set to “ENTREZID” and pAdjustMethod was set to “BH”.

### Cell-to-cell communication

Intercellular communication between distinct TDSCs clusters was calculated with the iTALK package (v0.1.0). The FindLR function loads genes that are either highly expressed or differentially expressed, mapping them to the ligand-receptor database.

The normal cells and diseased cells were cultured in the complete medium for 24 hours. The supernatants were individually collected and subsequently subjected to centrifugation at 100 g for a duration of 5 minutes. The initial medium of two new sets of healthy cells was discarded and substituted with the aforementioned supernatant. The cells were cultured for a further 24 hours at a temperature of 37°C in the presence of 5% CO2. The alterations in gene expression of TDSCs were observed in various supernatant environments.

### SiRNA transfection

Rat TDSCs was employed in this experiment. The TDSCs were seeded in 6-well plates at a density of 20,000 cells per well and subsequently incubated at 37°C in a CO2-controlled incubator. The progression of TDSCs was meticulously monitored through an inverted optical microscope, and the transfection process was initiated once the cells had attained a growth rate of 60% to 80%. Two sterile centrifuge tubes with a volume of 1.5ml was utilized for the following procedure. Lipofectamine^®^ RNAiMAX Reagent Transfection reagent and 7μl diluted siRNA solution (20μM) were respectively diluted with 150μl Opti-MEM^®^ serum-reducing medium in 1.5ml sterile centrifugal. The diluent for the transfection reagent was combined with the diluent for the siRNA in equal proportions to generate a system measuring 300μl. The mixed system was allowed to incubate for a duration of 5 minutes at ambient temperature. Following incubation, introduce a mixture of transfection reagent and siRNA into each well. The cells were relocated to a thermostatic incubator and were sustained for further growth.

### Wound healing assay

Rat TDSCs was employed in this experiment. The group subjected to experimentation received siPRDX2 transfection, while the control group was administered with an equivalent amount of control. Upon the completion of successful transfection, the growth of HOS cells was meticulously monitored through the use of an inverted microscope. Subsequently, wound healing assay was conducted once the cells had fully spread. The 6-well plates containing transfected TDSCs were positioned within a sterile work area. Utilizing the aseptic ruler as a guide, employ the 200μl aseptic gun head to create a perpendicular scratch on the bottom plate in a straight manner. It is imperative that the gun head remains level and does not tilt during the operation, while ensuring the scratch maintains a consistent width. To cleanse the cells, it is imperative to add 1 milliliter of sterile PBS buffer to each well. This action must be repeated three times to ensure complete washing. Afterward, it is necessary to remove the scratched suspended cell mass. The cells were subsequently transferred to a constant temperature incubator for continued cultivation. Wound healing was monitored through an inverted cell microscope at two different intervals, which were at the start (0h) and after 24 hours.

### Transwell assay

Rat TDSCs was employed in this experiment. The experimental cells underwent transfection with siPRDX2, whereas the control cells were administered an equivalent quantity of control. After a successful transfection, the cells were fully digested using pancreatic enzymes. Following this, the cells were resuspended after being counted to achieve a cell suspension of 50,000 cells/ml. The transwell chamber, which was equipped with matrix glue, was positioned on a pristine work surface, and a volume of 0.5ml of complete medium was introduced into the lower chamber. The upward chamber received a 200ul cell suspension. The transwell chamber was subjected to a constant temperature environment. Following a 24-hour incubation period, the cells were immobilized using a 4% neutral paraformaldehyde fixative for a duration of 30 minutes. Subsequently, they were subjected to crystal violet dye staining for a duration of 20 minutes. After extracting the residual cells from the semi-permeable membrane of the chamber, they were subjected to observation under an inverted microscope.

### Motif enrichment analysis

To identify residues in overlapping functional motifs, sequences with the co-accessibility regulatory were collated and uploaded to MEME Suite v.5.4.1 (a motif-based sequence analysis tool). The sequences were converted using the getSeq function in the Biostrings package. Motifs used for enrichment testing were obtained from the HOCOMOCO human (v11 CORE) database.

### Statistical analysis

All experiments were performed in triplicate. Statistical tests were performed *via* bilateral assessments. Statistical significance was accepted when P < 0.05. The biological experiments were conducted thrice to ensure reproducibility. Bar graphs in this paper represent the mean and error bars SD or SEM, the statistical difference between groups is indicated on graphs with stars: the stars (from 1-4 stars) respectively represent p-values less than 0.05, 0.01, 0.001 and 0.0001. R software version 4.0.2 (https://www.r-project.org/) was used for the analyses. Certain R packages, including Seurat, Monocle, GenomeInfoDb, ggplot2, Signac, iTALK, parallel, harmony, and karyoploteR, were used in this study.

## Results

### Single-cell landscape in tendinopathy

**Figure 1:**
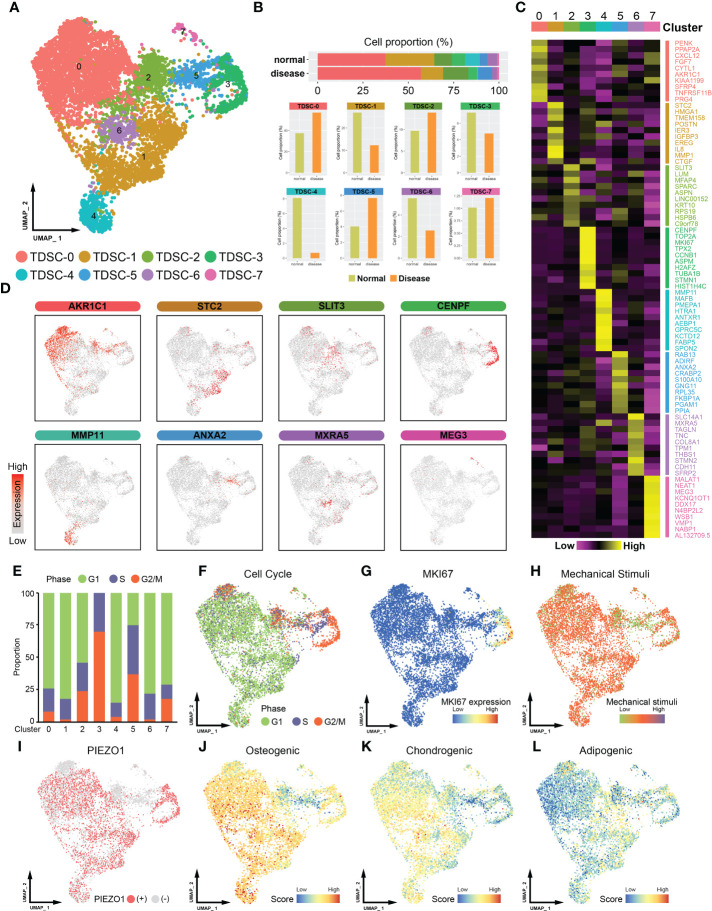
Single-cell RNA sequencing (ScRNA-seq) -based profiling of tendon microenvironments (the data was sourced from GSE150482). **(A)** A UMAP plot showing color-coded cell clusters in the tendon microenvironment. TDSCs can be divided into eight subgroups (from TDSC-0 to TDSC-7). **(B)** The distribution of healthy and tendinopathy cells in different clusters. The upper section of the map depicts the cumulative representation of cell subsets in disease or normal specimens. The bottom half is a detailed comparison of each cell subpopulation. **(C)** Heatmap showing marker gene expression in different cell clusters. The subcluster is indicated by the superscript numeral. The gene names that correspond to the given expression information are located on the right-hand side. **(D)** UMAP plot showing the expression levels of marker genes in cell clusters. **(E)** Histogram showing the proportions of different cluster cells in the G1, S or G2/M phase. **(F)** UMAP plot showing different cell cycle distributions. **(G)** *MKI67* expression distribution map. **(H)** UMAP plot showing the level of mechanical stimulation in cell clusters. **(I)** UMAP plot showing the expression distribution of Piezo1 in cell clusters. **(J)** Osteogenic differentiation capacity of the TDSCs. **(K)** Chondrogenic differentiation capacity of the TDSCs. **(L)** Adipogenic differentiation capacity of the TDSCs.

A total of 7380 TDSCs, including 2651 cells from normal tendon samples and 4729 cells from tendinopathy samples, were studied on the basis of scRNA-seq data (**Figures 1A**, **S1**, the data was sourced from GSE150482). Unbiased clustering of single-cell transcriptomes led to the recognition of 8 major clusters (from TDSC-0 to TDSC-7, **Figure 1B**). Cluster-specific markers were labeled and visualized; analysis of these marker genes allowed general identification of distinct clusters characteristics (**Figures 1C, D**). TDSC-0 cluster cells expressed high levels of *AKR1C1*, indicating that the TDSC-0 cluster may predominantly include cells that sense inflammatory stimuli. TDSC-1 cluster cells expressed relatively high levels of migratory cell markers (*STC2* and *HMGA1*). TDSC-2 cluster cells overexpressed genes related to fibrosis, such as *SLIT3* and *LUM*. TDSC-3 cluster cells, which expressed *CENPF* and *MKI67*, was associated with cell proliferation. TDSC-4 cluster cells showed anti-inflammatory ability (expressing *MMP11* and *FABP5*). Adipogenic differentiation regulators (*ADIRF*, *CRABP2* and *ANXA2*) were expressed in TDSC-5 cluster cells. TDSC-6 cluster cells were critical to extracellular matrix remodeling (expressing *MXRA5*). TDSC-7 cluster cells, which expressed *MALAT1* and *MEG3*, were associated with inhibited cell migration (the references are summarized in **Table S1**).

Cell cycle analysis showed that the TDSC-3 cell cluster contained a higher proportion of G2/M‐phase cells (**Figures 1E, F**). Consistent with this finding, cells in the TDSC-3 cluster expressed higher levels of *MKI67*, implying that they showed higher proliferative capacity (**Figure 1G**). Next, physiologically modest mechanical stimulation is often beneficial, stimulating healing and complete tendon formation (13, 14). *Piezo1*, a mechanosensitive ion channel, promotes tendon performance by enhancing tissue stiffness and strength (15, 16). We found that cells in the TDSC-2, TDSC-5 and TDSC-7 clusters were exposed to lower mechanical stimulation (**Figure 1H**). Consistently, Piezo1 was weakly expressed in TDSC-2, TDSC-5 and TDSC-7 cells, which meant that they were in different mechanical states (**Figure 1I**). In addition, to assess the stem cell features of different clusters, we characterized the three-way differentiation capacity of the TDSCs. Most of the cells exhibited high osteogenic and chondrogenic abilities; however, TDSC-2 and TDSC-5 cells showed a more pronounced adipogenic tendency (**Figures 1J-L**).

### Identification of diseased cells in tendinopathy

**Figure 2:**
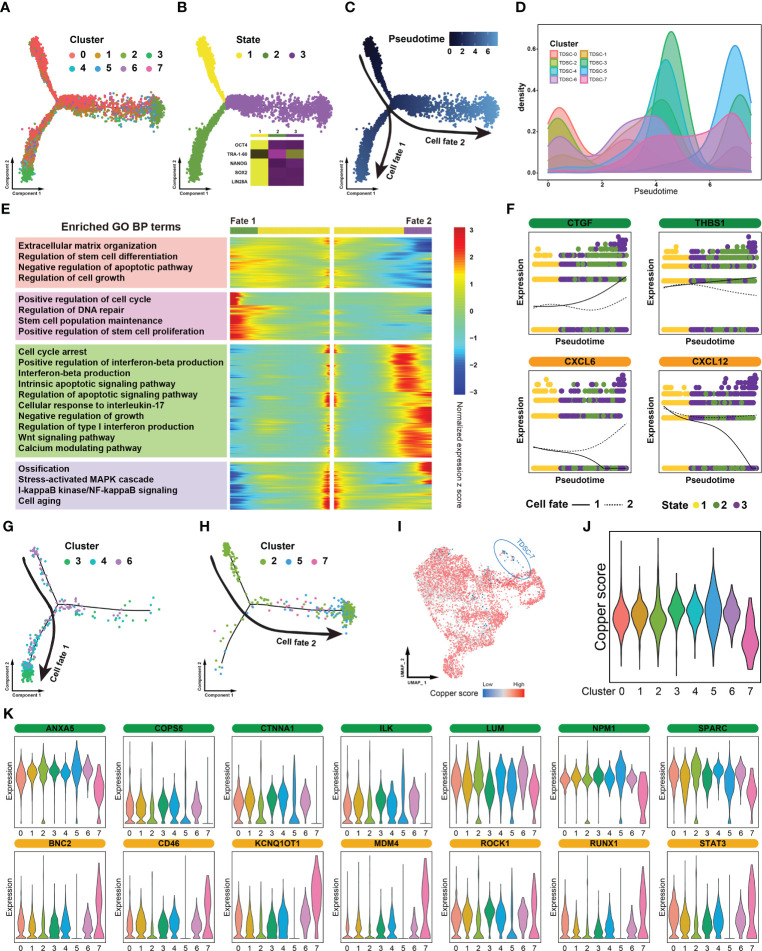
Identification of diseased cells in tendinopathy. **(A)** Pseudotime analysis of color-coded cells in distinct clusters. **(B)** Pseudotime analysis of cells in different states. The heat map situated in the lower right quadrant depicts the expression of stem cell markers across various cellular states. **(C)** Trajectory of cells at different pseudotime. **(D)** Cell density distribution map of distinct clusters in accordance with the differentiation trajectory. **(E)** Trajectory heatmap of different cell fates. The heat map is partitioned into four modules in accordance with the mode of the expression. The left portion of the image displays the results of the module enrichment analysis. **(F)** Separate branch curves showing the kinetic trend of crucial genes. The cellular fates are distinguished by the presence of either dashed or solid lines. **(G)** Cellular composition of the cell fate 1 group. **(H)** Cellular composition of the cell fate 2 group. **(I)** Distribution map of cuproptosis levels in different clusters. **(J)** Violin plot showing cuproptosis levels in distinct clusters. **(K)** Violin plot showing the expression levels of certain vital genes that affect tendon repair. The genes that facilitate tendon healing are depicted in green, while those that impede it are represented in yellow.

To reveal the dynamics and regulation of cell fate decisions in different TDSCs clusters, we performed a Monocle pseudotime analysis. TDSCs were divided into three pseudotime stages, and the different cell clusters were located in distinct regions of the trajectory (**Figure 2A**). Because they highly expressed certain classical progenitor/stem cell markers (*OCT-4*, *NANOG*, *SOX2* and *LIN28A*), the cells in state 1 were considered to be at the origin of the trajectory (**Figure 2B**). Along the differentiation trajectory, the cluster cell groups diverged into two separate paths, representing two distinctive cell fates (**Figure 2C**). TDSC-0 and TDSC-1 cells were located in the beginning pseudotime branch, while the others were mainly located in the terminus of the trajectory (**Figure 2D**).

“Branch-dependent” genes along the pseudotime trajectory were identified and assigned to four gene modules. A Gene Ontology analysis showed that some functions beneficial to tendon repair (such as extracellular matrix organization and positive regulation of stem cell proliferation) were significantly activated in the cell fate 1 group; however, certain functions critical tendinopathy exacerbation (such as cell cycle arrest and ossification) were enhanced in the cell fate 2 group (**Figure 2E**). When we focused on individual genes, the expression of certain restorative genes, such as *CTGF* and *THBS1*, was increased in the cell fate 1 group but decreased in cell fate 2 group. Moreover, some inflammation-related genes showed diametrically opposed expression patterns (e.g., *CXCL6* and *CXCL12*) (**Figure 2F**). Combining the results of these two analyses, the cells in the fate 1 group were identified as tendinopathy repair cells, and the cells in the fate 2 group were identified as promoters of disease progression. Through an in-depth analysis of the composition of the cells in the two fate groups, we found that the cell fate 1 group consisted mainly of TDSC-3, TDSC-4 and TDSC-6 cells and that the cell fate 2 group consisted of TDSC-2, TDSC-5 and TDSC-7 cells (**Figures 2G, H**).

Furthermore, a recently defined form of cell death, cuproptosis, is an essential and finely tuned process that is critical for the removal of damaged and superfluous cells. Therefore, a cuproptosis score map was plotted on the basis of uniform manifold approximation and projection (UMAP) dimensionality reduction. The majority of the cells exhibited modest cuproptosis rates, but not the TDSC-7 cells, in which it was inhibited (**Figures 2I, J**). Moreover, TDSC-7 cells showed lost expression of numerous tendon-healing genes and enhanced expression of many biomarkers related to promoted tendon injury (**Figure 2K**). In summary, these results suggest that TDSC-7 cells, comprising a specialized cell population, exhibited extremely abnormal cuproptosis rates. We therefore proposed that TDSC-7 be termed diseased cells in tendinopathy.

### PRDX2 expression is low in TDSC-7 and is correlated with tendinopathy progression

**Figure 3:**
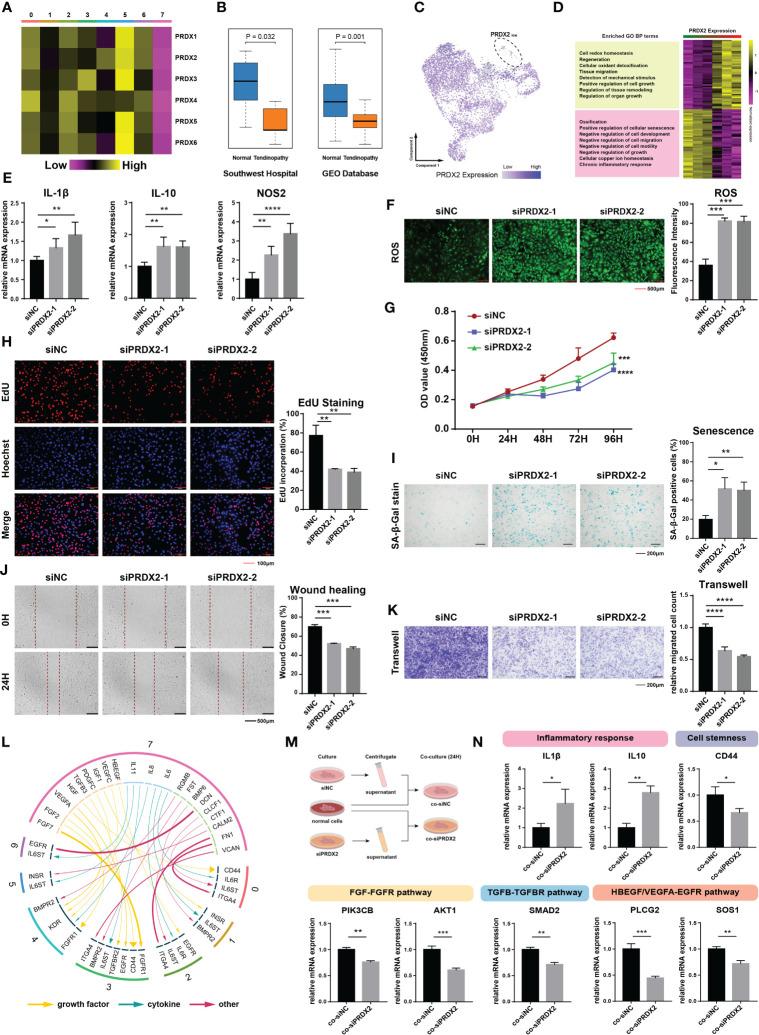
Expression and biological function of PRDX2. **(A)** Heatmap plot showing the expression patterns of peroxiredoxin family genes in distinct cluster cells. **(B)** The boxplot showing a discernible discrepancy in the expression of *PRDX2* between tendinopathy and normal tendons in both the GEO cohort (n=46) and the Southwest hospital cohort (n=10). **(C)** *PRDX2* expression distribution map of different clusters. **(D)** Heatmap plot showing the gene ontology difference between cells with high- and low- *PRDX2* expression TDSCs. The left portion of the image displays the results of the module enrichment analysis. **(E)** *PRDX2* silencing enhanced the expression of inflammatory genes. Each group perform three repetitions. **(F)** *PRDX2* silencing increased intracellular reactive oxygen species (ROS) levels. **(G)** cell counting kit-8 (CCK-8) assays were performed to assess the cell proliferative capacity after *PRDX2* silencing. **(H)** EdU staining was performed to evaluate the cell proliferation rate after *PRDX2* silencing. **(I)** β-Gal staining was performed to assess the senescence of *PRDX2*-silenced TDSCs. **(J)** Wound healing assays were performed to evaluate the migratory capacity of *PRDX2*-silenced TDSCs. **(K)** Transwell assays were performed to assess the migratory capacity of *PRDX2*-silenced TDSCs. **(L)** Circos plot showing cellular crosstalk between diseased cells and cells in other clusters. The yellow arrows represent “growth factor” categories, the green arrows represent “cytokine” categories and the red arrows represent “other” categories. The degree of thickness exhibited by the arrow is indicative of the intensity of the interaction. The numeral inscribed on the outermost circumference and the hue of the circular band both denote the subgroup of cells that they correspond to. **(M)**. Flow chart showing the verified intercellular communication network. **(N)**. PCR validated the inferred cell–cell communication results. The symbols *, **, *** and **** respectively represent p-values less than 0.05, 0.01, 0.001 and 0.0001.

To determine the key molecular mechanism, we meticulously analyzed the gene expression patterns of diseased cells. We found that peroxiredoxin family genes, including *PRDX1* to *PRDX6*, were all under expressed in TDSC-7 (**Figure 3A**). Two sets of microarray data in tendinopathy were additionally validated, one from our previous study (Southwest Hospital cohort) and the other from the Gene Expression Omnibus (GEO) database (GSE26051 dataset). Since only PRDX2 was expressed less in tendinopathy than in control normal tendon tissue in both the microarray cohorts and that it was expressed in almost all TDSCs, except the TDSC-7 cluster cells, we hypothesized that *PRDX2* is a potential specificity marker and an ideal target for diseased cells (**Figures 3B, C**). Gene pattern differences were compared between the 1500 cells with the highest *PRDX2* expression and the 1500 cells with the lowest *PRDX2* expression. A Gene Ontology analysis showed that high *PRDX2* expression contributed to cell redox homeostasis and regeneration, whereas low *PRDX2* expression was mainly associated with cellular senescence, and negative regulation of cell growth and migration (**Figure 3D**).

*PRDX2* encodes a member of the peroxiredoxin family of antioxidant enzymes that regulates diverse cellular functions, including oxidative stress, cell proliferation, migration and senescence (17–19). We speculate that *PRDX2* plays a crucial role in the biological effects of diseased cells. Rat TDSCs was utilized to validate the biological activity of PRDX2. Silencing *PRDX2* significantly increased the expression of certain proinflammatory factors (such as *IL-1β*, *IL-10* and *NOS2*) in the TDSCs (**Figures 3E**, **S2A, B**). In addition, intracellular reactive oxygen species (ROS) levels were significantly enhanced in PRDX2-silenced cells (**Figure 3F**). We used cell counting kit-8 (CCK-8) and 5-ethynyl-2′-deoxyuridine (EdU)-staining assays to assess the proliferative effect of siRNA transfection on the TDSCs. Forty-eight hours after siRNA transfection, the proliferation of *PRDX2*-silenced tendon cells was significantly attenuated (**Figure 3G**). The proportion of EdU-positive proliferating *PRDX2*-silenced TDSCs was significantly reduced (**Figure 3H**). The proportion of senescent cells was markedly increased in the *PRDX2*-silenced TDSCs population (**Figure 3I**). Moreover, the migration of TDSCs is critical for the regenerative healing of tendinopathy (20). Wound healing assays confirmed the gap in the monolayer consisting of TDSCs with *PRDX2* silenced closed more slowly than that in the monolayer consisting of normal cells (**Figure 3J**). Similar results were observed in transwell assays, showing that fewer highly mobile cells in the *PRDX2*-silenced group, as indicated by the number of cells that passed through the membrane insert (**Figure 3K**). All these results confirmed that diseased cells with low *PRDX2* expression maintained a worse cellular state, hindering tendinopathy recovery.

We wondered about the influence of diseased cells on the tendon microenvironment. Therefore, a cell–cell communication analysis was performed, and ligand–receptor interactions between diseased cells and cells in other clusters were visualized (**Figure 3L**). Certain inflammatory factors (*IL6*, *IL11*, *CLCF1* and *CTF1*) secreted by diseased cells interacted with *IL6ST* and *IL6R*, which were highly expressed on the surface of cells in other clusters, inducing their inflammation. HGF secreted by diseased cells regulated peripheral cell stemness by affecting *CD44*. Furthermore, many other complex and tight interactions, such as FGF-FGFR, TGFB-TGFBR, and HBEGF/VEGFA-EGFR, affected the tendon microenvironment. We performed qPCR to validate the cell–cell communicating pairs that we had preliminarily identified (**Figure 3M**). As expected, cells cultured in diseased cell supernatant showed higher levels of inflammation, decreased cell stemness, and significantly inhibited activation of healing-related pathways such as the *FGFR*, *TGFBR*, and *EGFR* pathway (**Figures 3N**, **S2D**).

### The transcription factor FOXO1 promotes PRDX2 expression

**Figure 4:**
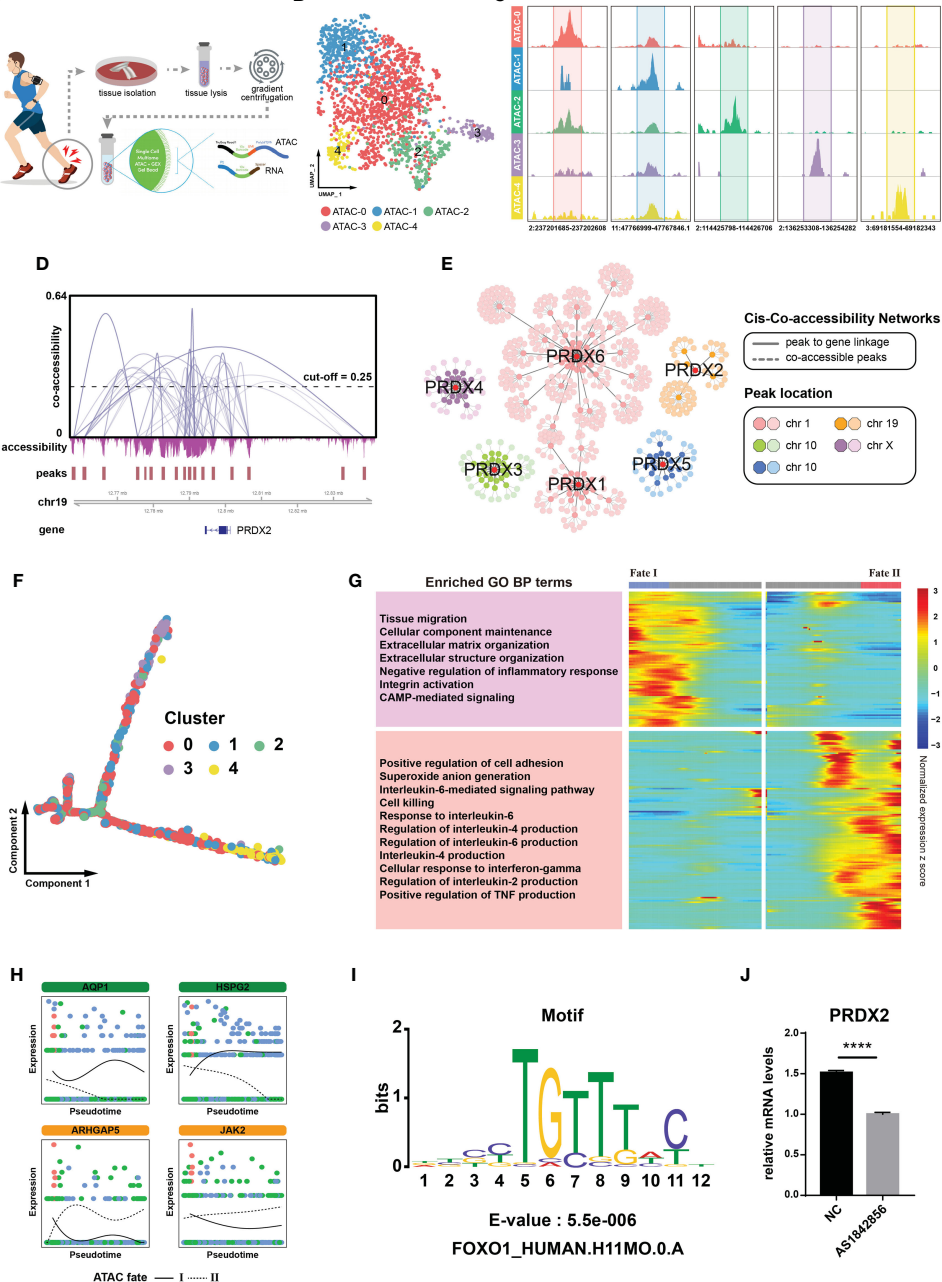
FOXO1 regulates PRDX2 transcription. **(A)** Flow chart showing single-cell multi-modal ATAC and gene expression sequencing process. **(B)** UMAP plot showing different cell clusters in the multi-modal ATAC and gene expression sequencing analysis. Distinctive colours are utilized to represent various cell clusters. **(C)** Chromatin accessibility plot showing mark sites in distinct cell clusters. The upper peaks denote the accessibility of chromatin. The numerical value at the base denotes the location of the chromatin. **(D)** Cicero co-accessibility around *PRDX2* position. The gray curve connecting the two peaks indicates a correlation in accessibility. The horizontal dashed line serves as an indicator for the cut-off point of co-accessibility. **(E)** Co-accessibility regulatory network of the peroxiredoxin family. The correlation between peak accessibility and gene expression is depicted by the gray solid line. The co-accessibility between peaks is illustrated by the gray dotted line. **(F)** Pseudotemporal trajectory of distinct ATAC cluster cells. **(G)** Trajectory heatmap showing different ATAC cluster cell fates. The left portion of the image displays the results of the module enrichment analysis. **(H)** Separate branch curves showing the kinetic trend of crucial genes in the ATAC trajectory. The cellular fates are distinguished by the presence of either dashed or solid lines. **(I)** *FOXO1* motif was enriched in the *PRDX2* region. **(J)** PCR showing that *PRDX2* expression was reduced after the addition of FOXO1 inhibitors. The symbol **** represent p-values less than 0.0001.

To characterize the upstream regulatory landscape in diseased cells, we performed single-cell multi-modal ATAC and gene expression sequencing in tendinopathy, which enabled combined profiling of accessible chromatin and RNA within the same cell (21) (**Figure 4A**). Based on chromatin accessibility patterns, 1965 tendinopathy cells were found in 5 cell clusters by unsupervised clustering (from ATAC-0 to ATAC-4, **Figures 4B, C**). We investigated the cis-regulatory mechanism of the peroxiredoxin family. Since chromatin accessibility data is extremely sparse, the Cicero algorithm was used to optimize for denser count data and provide an accurate estimate of co-accessibility. We found many interactions between peaks near the peroxiredoxin family positions (**Figures 4D**, **S3A**). By integrating snRNA-seq data, peak-to-gene linkages were also identified to look for correlations between peak accessibility and gene expression. Furthermore, a co-accessibility regulatory network was generated (including 550 peak-peak and 132 peak-gene interactions, **Tables S2, 3**), that clearly delineated cis-regulatory connections of the peroxiredoxin family (**Figure 4E**).

Then, we performed a pseudotime analysis to verify the cell fates described above. The Monocle algorithm resulted in a similar “root-trunk”-like trajectory as that identified through clustering, starting from the root and gradually differentiating into two distinct cell fates (**Figures 4F**, **S3B**). The cell density distribution map showed that ATAC-0, ATAC-1 and ATAC-2 were mainly located in the initial portion of the trajectory, while ATAC-3 and ATAC-4 were mainly located at the end (**Figure S3D**). Different cell fates awaited distinct cell clusters; for example, ATAC-4 mainly followed cell fate I, while ATAC-3 predominantly followed cell fate II (**Figure S3D**). We examined the expression pattern of *PRDX2* in these clusters; interestingly, *PRDX2* was negligibly expressed in the ATAC-3 cluster cells (**Figure S3E**). Trajectory heatmaps helped to further annotate the cell states. Certain processes that contributed to tendinopathy recovery (e.g., extracellular matrix organization) were significantly activated in cell in the ATAC fate I group, whereas other processes, such as those that contributed to disease progression, were significantly enhanced in the ATAC fate II group (**Figure 4F**). Cells in the ATAC fate I group showed higher expression of *AQP1* (an anti-senescence gene (22)) and *HSPG2* (a collagen-related gene (23)), whereas cells in the ATAC fate II group expressed higher levels of *ARHGAP5 *(24) and *JAK2 *(25), which inhibit stem cell activity (**Figure 4G**). That is, cells in the ATAC fate I group (mainly consisting of ATAC-4 cells) were identified as tendinopathy-repairing cells, corresponding to the previously determined characteristics of the cell fate 1 group, and cells in ATAC fate II group (mainly consisting of ATAC-3 cells) promoted disease progression, corresponding to the previously determined characteristics of the cell fate 2 group. According to these analysis results, cells in ATAC-3 cluster group were diseased cells.

We revealed the chromatin accessibility within 2.5 kilobases of the *PRDX2* transcription site (**Figure 4H**). A motif analysis was performed to identify factors acting on the co-accessibility regulatory network and found that the *FOXO1* motif was significantly enriched in the sequences (**Figure 4I**). We carried out PCR to test the hypothesis suggesting that the *FOXO1* motif is related to *PRDX2* expression in tendinopathy. We treated cells with AS1842856, which reduces FOXO1 activity by specifically binding it (26). We found that *PRDX2* expression was significantly reduced in the TDSCs treated with AS1842856 (**Figure 4J**). Therefore, FOXO1 was confirmed to be a potential upstream regulator of PRDX2.

### PRDX2 effects tendinopathy pathogenesis by targeting the TNF signaling pathway

**Figure 5:**
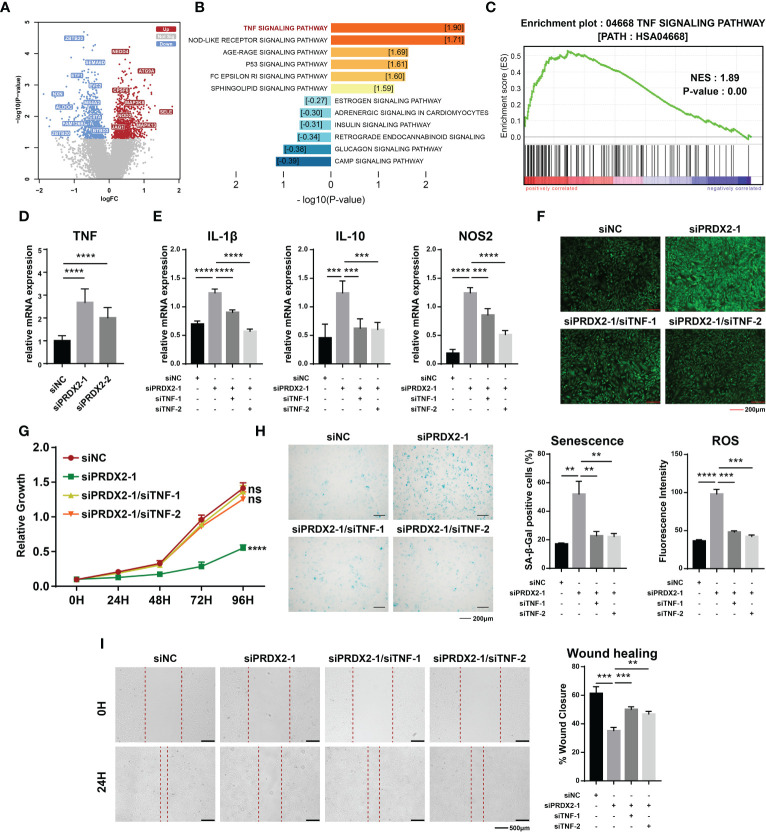
The TNF signalling pathway is downstream of PRDX2. **(A)** Differentially expressed genes in the low-*PRDX2* expression patient group. Genes that are characterized by increased expression in the low-expression PRDX2 group are denoted by the color red, while those with decreased expression are indicated by the colour green. **(B)** Enrichment analysis of differentially expressed genes. The activation of a pathway is symbolized by warm colours, while the suppression of the pathway is represented by cool colours. The numbers in the box represent Normalized Enrichment Score. **(C)** GSEA enrichment analysis showing the TNF signalling pathway was significantly activated in patients with low *PRDX2* expression. **(D)** PCR confirmed that *PRDX2* silencing resulted in enhanced TNF expression. **(E)** PCR results showing that TNF inhibition significantly reversed the increase in inflammatory markers caused by *PRDX2* silencing. **(F)** TNF inhibition reversed the increase in intracellular ROS in *PRDX2*-silenced cells. The statistical results are shown on the below. **(G)** Cell counting kit-8 (CCK-8) assay results showed that TNF silencing reversed the inhibition of cell proliferation induced by *PRDX2* silencing. **(H)** TNF inhibition alleviated TDSCs senescence. The statistical results are shown on the right. **(I)** Wound healing showing that TNF inhibition reversed the diminished migration of *PRDX2*-silenced cells. The symbols **, *** and **** respectively represent p-values less than 0.01, 0.001 and 0.0001.

To further investigate the functional mechanism of *PRDX2*, we integrally analyzed the microarray data related to tendons (GSE26051). The expression data on fifty-six patients were divided into high or low expression groups according to the median value of *PRDX2* expression. In the low-*PRDX2* expression group, the expression of 827 genes was significantly increased, and that of 680 genes was decreased (**Figure 5A**). A gene set enrichment analysis (GSEA) of differentially expressed genes showed that the TNF signaling pathway was dramatically activated in the low-*PRDX2* expression group (**Figures 5B, C**). Hence, we assumed that *PRDX2* regulated TDSCs by targeting the TNF signaling pathway. To verify this hypothesis, the expression levels of TNF were detected after *PRDX2* silencing. As expected, *PRDX2* depletion led to a marked increase in TNF expression in the TDSCs (**Figure 5D**).

Rescue experiments were performed to characterize the regulatory relationship. We explored the effect of putative downstream factors on the level of inflammation in TDSCs and found that inhibition of TNF signaling significantly reversed the increased expression of *IL-1β*, *IL-10* and *NOS2* caused by *PRDX2* silencing (**Figure 5E**). In line with this finding, TNF silencing significantly reversed the increase in intracellular ROS in *PRDX2*-silenced cells (**Figure 5F**). Similarly, inhibition of the proliferation and aging of the TDSCs induced by *PRDX2* silencing was attenuated by TNF signaling inhibition (**Figures 5G, H**). Additionally, cell migration had been shown to be impaired after *PRDX2* silencing, and then, we demonstrated that cell migration was restored after TNF signaling inhibition (**Figure 5I**). Taken together, our findings suggest that diseased cells contribute to tendinopathy pathogenesis by activating the TNF signaling pathway.

## Discussion

Tendinopathy describes a chronic disease that affects damaged and diseased tendons characterized by pain and reduced function (27). Herein, we mapped the cellular landscape of human tendinopathy using single-cell genomics and identified eight cell populations with distinct functions in the microenvironment. In the past, noninflammatory or degenerative perspectives dominated theories explaining tendinopathy pathogenesis. While, Rees et al. demonstrated that the inflammatory response is a key component in chronic tendinopathy (28). Our study supported this notion that the TDSC-0 cells, comprising the largest cell subset in damaged tendons, overexpressed the inflammation-related genes AKR1C1 and CFD.

The self-renewal capacity of TDSCs is critical for tendinopathy healing and tendon regeneration. Previous studies have shown that in physiological niches, most TDSCs are in a quiescent state, and when cells are exposed to an external stimulus, such as mechanical loading, inflammation and biological factors, certain TDSCs are activated and begin to self-renew or differentiate (29). Consistent with this previous finding, our study revealed that the cell cycle was inactive in most TDSCs, and it was only markedly activated in the TDSC-3 cells. This outcome was confirmed by the expression of *MKI67* by the TDSC-3 cells. Ardem Patapoutian, a 2021 Nobel Laureate in Physiology or Medicine, discovered that Piezo1 is an essential receptor for mechanical stimulation (30). Both mechanical stimulation scores and Piezo1 expression confirmed that TDSC-2, TDSC-5 and TDSC-7 are in a stress-insufficient state, which might be the cause of slow and frequently poor tendon healing. TDSCs are stem cells with classical mesenchymal stem cells (MSCs) characteristics, including osteogenic, chondrogenic, and adipogenic differentiation capacities (31, 32). Erroneous differentiation of TDSCs may contribute to the pathogenesis of chronic tendinopathy (33). Our differentiation capacity analysis revealed that different cell subsets exhibited various differentiation propensities, confirming that different subsets play distinct roles in tendinopathy progression.

Upon close inspection of the cell proportion alterations between normal and disease tendon samples, it was observed that the proportion of cells belonging to TDSC-1, -4, and -6 demonstrated a significant decrease in the tendinopathy sample, whereas TDSC-5 exhibited an increase. These proportional trends observed are in accordance with the distinct roles played by various cell subsets. The findings of our study suggest that TDSC-1, -4, and -6 are associated with the functions of cell migration, anti-inflammatory response, and extracellular matrix remodeling, respectively. The dysfunction of these physiological processes constitutes the crucial element that drives the progression of tendinopathy. TDSC-5 exhibits an atypical potential of three-way differentiation. Increased TDSC-5 leads to the abnormal differentiation of stem cells, which is a distinctive feature of tendinopathy (34).

The Monocle algorithm was used to analyze the transcriptional dynamics of the TDSCs in this study, revealing details in cell differentiation fates. Specifically, in the cell fate 1 group, the proliferative capacity of the stem cells was significantly enhanced, and consistent with the results indicating their cell cycle activation, hyperproliferative TDSC-3 mainly followed the cell fate 1. In a previous study, the ossification level among cells following the cell fate 2 was found to be increased, often leading to calcific tendinitis (35). Additionally, *MAPK* has been previously shown to be a crucial pathway in tendinopathy progression (36) and, in our study, was also significantly activated in the cell fate 2 group. Furthermore, *CTGF*, has been reported to induce the differentiation and proliferation of TDSCs (37), and *THBS1* promote has been shown to induce new fiber formation in injured tendons (38). The expression of both the *CTGF* and *THBS1* genes was decreased in the cell fate 1 group and increased in the cell fate 2 group. These results indicated that the two identified differentiation trajectories represented two distinct cell fates.

Cuproptosis analysis indicated that most of the TDSCs, including hyperproliferative cells (TDSC-3), maintained suitable cuproptosis levels. However, the cuproptosis level of the TDSC-7 cluster cells was abnormally decreased, indicating that these cells were in a precarious state. Copper is an essential trace metal, and proper copper supplementation has a beneficial effect on tendon regeneration and self-renewal (39). Herchenhan et al. found that an appropriate copper concentration was crucial for maintaining lysyl oxidase activity, which was required for orderly collagen fibril formation in tenocytes (40). Moreover, moderate intracellular copper concentrations maintain suitable cuproptosis homeostasis, which is critical for the removal of damaged and redundant cells in tendinopathy. Incongruous cuproptosis levels, either too high or too low, are thus detrimental; cuproptosis levels that are too high cause excessive cell death, and cuproptosis levels that are too low hinder tendon tissue repair (41). These results suggested that TDSC-7 cells were incapable of repairing injured tendons.

Admittedly, the proportion of TSC-7 in the tendon is relatively small compared to other subclusters. However, we did observe that TDSC-7 displayed distinct features in relation to cell differentiation fates, cuproptosis levels, and the expression of certain crucial genes, suggesting that TDSC-7 cells were incapable of repairing tendinopathy. Furthermore, the cell–cell communication analysis indicates that TDSC-7 has the ability to modify the microenvironment of tendons by releasing certain cytokines that regulate the degree of inflammation and cellular stemness in adjacent subclusters of cells. Thus, TDSC-7 can be likened to a seed cell that triggers the progression of the disease. Similar theoretical models have been observed in other ailments, including gastric cancer and diabetes (42, 43). Furthermore, it was observed that the tendinopathy sample exhibited a rise in the proportion of TDSC-7; however, the extent of the alteration was not as substantial as in the remaining subclusters, which may be related to the heterogeneity among the specimens. This phenomenon has also been observed in previous authoritative literature. Zhang and his colleagues found that the proportion of cluster 3, which is favorable to disease recovery, was found to be also relatively low, with just a slight rise in the 3D-cultured recovery group (44).

*PRDX2*, a member of the peroxiredoxin family of antioxidant enzymes, has been identified as a signaling in diseased cells (TDSC-7) in tendinopathy. In fact, this gene has been shown to play a crucial role in many chronic diseases. Park et al. showed that PRDX2 deficiency led to increased endogenous H2O2, thereby exacerbating atherosclerosis (45). In addition, *PRDX2* has been shown to be required for insulin secretion and insulin-dependent regulation of longevity (46). In our present study, we revealed that silencing *PRDX2* expression affected multiple TDSCs functions, including inflammation, proliferation, senescence and migration. Chronic inflammation is a feature of tendinopathy (47), and anti-inflammatory treatments, including nonsteroidal anti-inflammatory drugs (NSAIDs) and topical glucocorticoids, are used in clinical therapy (48). Our study showed that *PRDX2* silencing in TDSCs increased proinflammatory factor expression and intracellular ROS levels, promoting damage progression. Additionally, high-proliferative capacity and youthfulness are key indicators of stem cell viability, guaranteeing their regenerative potential and therapeutic efficacy (49, 50). However, *PRDX2* silencing resulted in stem cell proliferation arrest and severe senescence. In addition, reduced expression of *PRDX2* concomitantly impaired certain other healing-related processes, such as migration. In summary, *PRDX2* was to blame for the deterioration of diseased cells (TDSC-7) in tendinopathy.

Single-cell multi-modal ATAC and gene expression sequencing is among the most recently developed technological solutions, enabling simultaneous profiling of the transcriptome and epigenome in the same cell. To the best of our knowledge, this is the first time ATAC and gene expression sequencing has been applied to dissect the mechanism of tendinopathy. Through in-depth analysis of multi-modal sequencing data, we confirmed the presence of diseased cells in tendinopathy. Moreover, we identified *FOXO1*, a member of the forkhead family of transcription factors, as an upstream regulator of *PRDX2* expression. In previous studies, *FOXO1* has been identified as a potential inhibitor of fibrosis capable of resisting oxidative stress and enhancing cell viability (51, 52). Our study suggested that FOXO1 affected tendinopathy progression by regulating *PRDX2* transcriptional activity. Further research indicated that TNF is a downstream pathway of *PRDX2* function, and we confirmed this speculation through corresponding rescue experiments. Moreover, Mokber et al. demonstrated that TNF plays an important role in the initiation of tendinopathy (53), and our study further elucidated the regulatory mechanism of TNF and deepened our understanding of tendinopathy.

In summary, our study confirmed the presence of diseased cells in tendinopathy, showing that these cells promote disease progression, influence the tendon microenvironment and curb disease recovery. PRDX2 is a crucial gene and may be a potential target in precision therapy.

## Data availability statement

Raw data of ordinary scRNA-seq can be accessed in the GEO (GSE150482), which was utilized to identify diseased cells in tendinopathy (54). Microarray data for tendinopathy can be found with GSE26051, this data was used to assess the expression levels of the peroxiredoxin family between tendinopathy and normal tissues (55). The single-cell multi-modal ATAC and gene expression sequencing data from Southwest Hospital are available in GEO database (GSE213803). The software and packages used to analyze the dataset are freely available.

## Ethics statement

The studies involving human participants were reviewed and approved by the Ethics Committee of the First Affiliated Hospital of Army Medical University, PLA. The patients/participants provided their written informed consent to participate in this study.

## Author contributions

Conception and design: KT, TL and BZ. Development of methodology: JG (1st author), XK and ZS. Analysis and interpretation of data: JG (1st author), HT, PH, CT and JG (7th author). Statistical analysis: XY, XK, JG (7th author), YS and ZS. Drafting of the manuscript: JG (1st author), JG (7th author) and TL. Critical revision of the manuscript: KT and BZ. Obtained funding: KT and TL. All authors contributed to the article and approved the submitted version.
