# Supplementary material for: Integrative single-cell RNA and ATAC sequencing reveals that the FOXO1-PRDX2-TNF axis regulates tendinopathy
Source: Front Immunol. 2023 May 8;14:1092778. doi: 10.3389/fimmu.2023.1092778 (PMC10200929; doi:10.3389/fimmu.2023.1092778)
Supplement: Supplementary file 1 [file DataSheet_1.docx]

Supplementary Material

**Supplementary Figure 1.** Quality control information. A. scRNA-seq quality control information. B. Quality control information of single-cell multi-modal RNA-seq. C. Quality control information of single-cell multi-modal ATAC-seq.


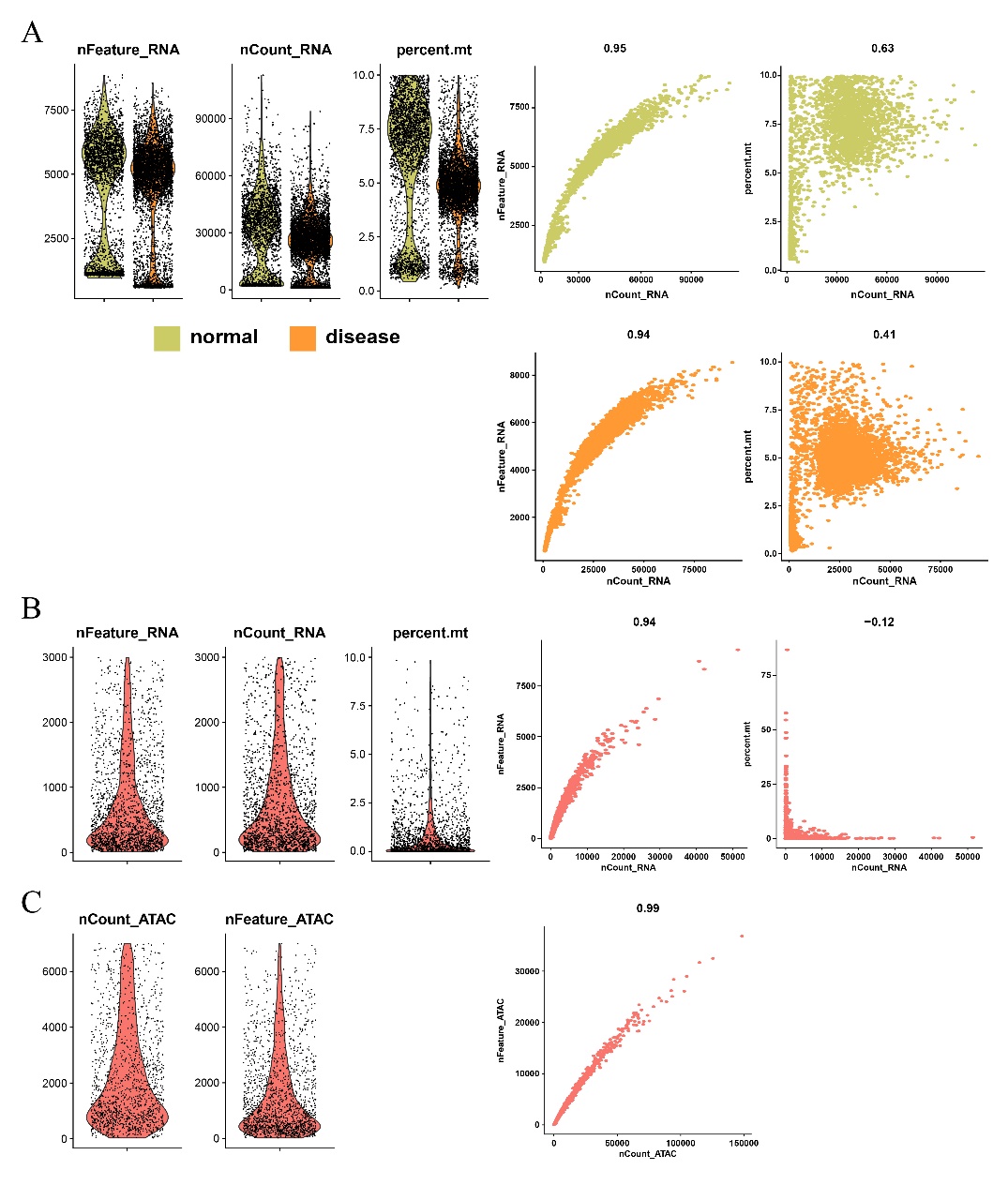


**Supplementary Figure 2. Expression and biological function of PRDX2. A.** The expression of PRDX2 was effectively silenceed with by siRNA. **B.** The levels of IL-1b and IL-10 increased in the supernatant. **C.** The level of apoptosis in cells did not exhibit a significant increase subsequent to the silencing of PRDX2. **D.** Western blot validated the inferred cell–cell communication results.


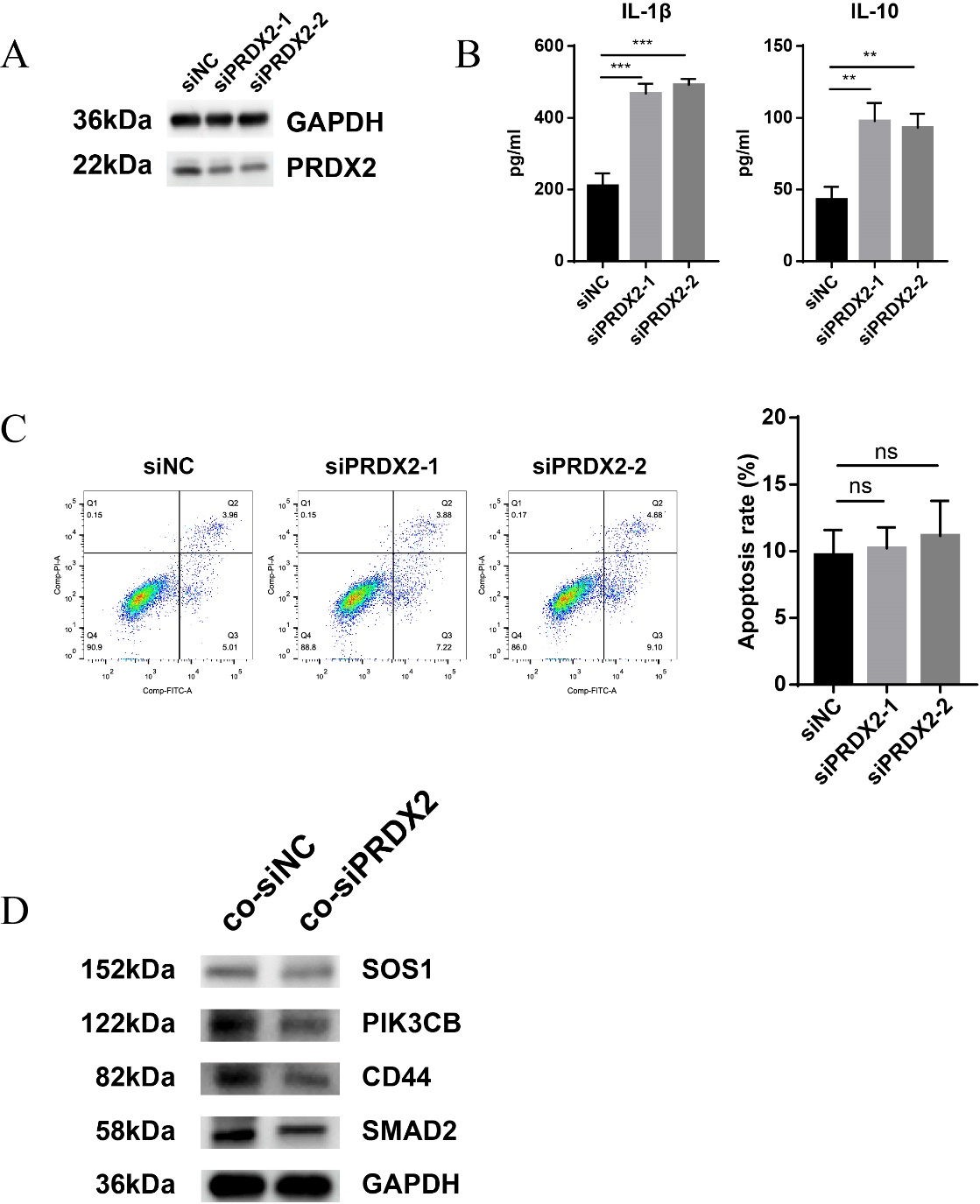


**Supplementary Figure 3.** The transcription factor FOXO1 promotes PRDX2 expression. **A.** Cicero co-accessibility around peroxiredoxin family. **B.** Pseudotime of ATAC cluster cells. **C.** Cell density distribution map of distinct ATAC cluster cells. **D.** Cellular composition of TDSCs following different trajectory fates. **E.** Heatmap showing PRDX2 expression in different ATAC cluster cells. **F.** PCR showing that *PRDX2* expression was reduced after FOXO1 silencing.


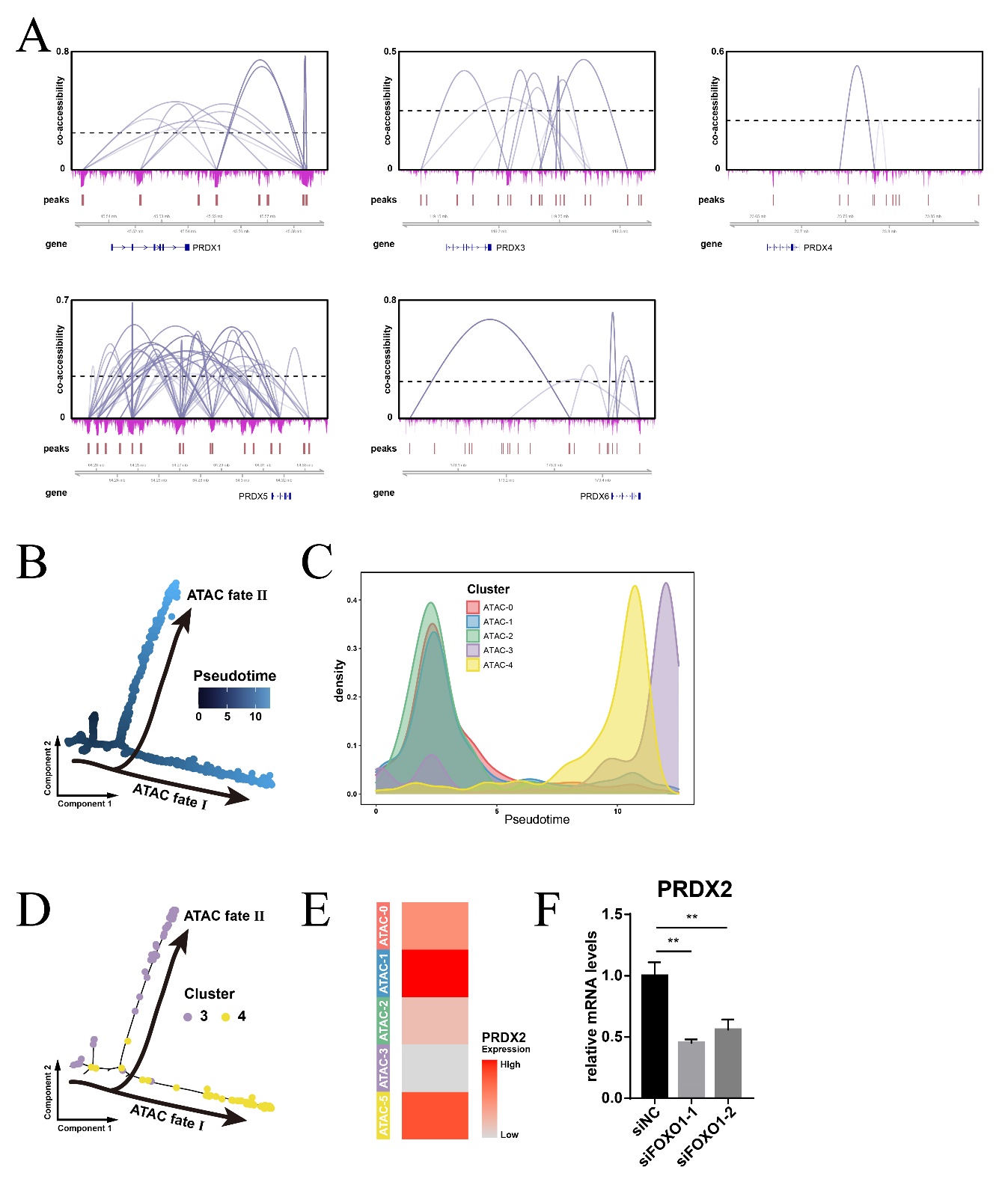


**Supplementary Table 1.** The specific markers for the 8 cell clusters in tendon tissues.

| cluster identity | marker genes | References |
| --- | --- | --- |
| 0 | AKR1C1, CFD | [1,2] |
| 1 | STC2, HMGA1 | [3,4] |
| 2 | SLIT3, LUM | [5,6] |
| 3 | CENPF, MKI67 | [7,8] |
| 4 | MMP11, FABP5 | [9,10] |
| 5 | ADIRF, CRABP2, ANXA2 | [11-13] |
| 6 | MXRA5, ACAN | [14, 15] |
| 7 | MALAT1, MEG3 | [16,17] |

This table is generated from the public literature cited.

**Supplementary References**

1. Wang, H.W., et al., *Reversal of inflammation-associated dihydrodiol dehydrogenases (AKR1C1 and AKR1C2) overexpression and drug resistance in nonsmall cell lung cancer cells by wogonin and chrysin.* Int J Cancer, 2007. **120**(9): p. 2019-27.

2. Chen, J., et al., *Complement factor D as a predictor of Achilles tendon healing and long-term patient outcomes.* Faseb j, 2022. **36**(6): p. e22365.

3. Qie, S. and N. Sang, *Stanniocalcin 2 (STC2): a universal tumour biomarker and a potential therapeutical target.* J Exp Clin Cancer Res, 2022. **41**(1): p. 161.

4. Lau, K.M., et al., *Overexpression of HMGA1 deregulates tumor growth via cdc25A and alters migration/invasion through a cdc25A-independent pathway in medulloblastoma.* Acta Neuropathol, 2012. **123**(4): p. 553-71.

5. Gong, L., et al., *SLIT3 deficiency attenuates pressure overload-induced cardiac fibrosis and remodeling.* JCI Insight, 2020. **5**(12).

6. Jepsen, K.J., et al., *A syndrome of joint laxity and impaired tendon integrity in lumican- and fibromodulin-deficient mice.* J Biol Chem, 2002. **277**(38): p. 35532-40.

7. Mahmoud, A.D., et al., *The Human-Specific and Smooth Muscle Cell-Enriched LncRNA SMILR Promotes Proliferation by Regulating Mitotic CENPF mRNA and Drives Cell-Cycle Progression Which Can Be Targeted to Limit Vascular Remodeling.* Circ Res, 2019. **125**(5): p. 535-551.

8. Hou, Y.Y., et al., *MicroRNA-519d targets MKi67 and suppresses cell growth in the hepatocellular carcinoma cell line QGY-7703.* Cancer Lett, 2011. **307**(2): p. 182-90.

9. Peruzzi, D., et al., *MMP11: a novel target antigen for cancer immunotherapy.* Clin Cancer Res, 2009. **15**(12): p. 4104-13.

10. Bogdan, D., et al., *Fatty acid-binding protein 5 controls microsomal prostaglandin E synthase 1 (mPGES-1) induction during inflammation.* J Biol Chem, 2018. **293**(14): p. 5295-5306.

11. Ni, Y., et al., *A Novel pro-adipogenesis factor abundant in adipose tissues and over-expressed in obesity acts upstream of PPARγ and C/EBPα.* J Bioenerg Biomembr, 2013. **45**(3): p. 219-28.

12. Petrie, K., et al., *Retinoic acid receptor γ is a therapeutically targetable driver of growth and survival in prostate cancer.* Cancer Rep (Hoboken), 2020. **3**(6): p. e1284.

13. Hogarth, M.W., et al., *Fibroadipogenic progenitors are responsible for muscle loss in limb girdle muscular dystrophy 2B.* Nat Commun, 2019. **10**(1): p. 2430.

14. Robins, J.E. and A.A. Capehart, *Matrix remodeling associated 5 expression in trunk and limb during avian development.* Int J Dev Biol, 2018. **62**(4-5): p. 335-340.

15. Wang, Y., et al., *Aspirin inhibits inflammation and scar formation in the injury tendon healing through regulating JNK/STAT-3 signalling pathway.* Cell Prolif, 2019. **52**(4): p. e12650.

16. Sun, Y. and L. Ma, *New Insights into Long Non-Coding RNA MALAT1 in Cancer and Metastasis.* Cancers (Basel), 2019. **11**(2).

17. Zhao, Y., et al., *Long non-coding RNA MEG3 regulates migration and invasion of lung cancer stem cells via miR-650/SLC34A2 axis.* Biomed Pharmacother, 2019. **120**: p. 109457.

**Supplementary Table 2.** The co-accessibility of peaks.

| Peak1 | Peak2 | co-access |
| --- | --- | --- |
| chr10-126397555-126398657 | chr10-126421582-126422473 | 0.346127536 |
| chr1-206495431-206496282 | chr1-206630872-206631772 | 0.337434803 |
| chr1-235908574-235909512 | chr1-235504183-235505072 | 0.701144558 |
| chr1-235908574-235909512 | chr1-235624528-235625437 | 0.401833992 |
| chr1-235908574-235909512 | chr1-235681096-235681981 | 0.393535567 |
| chr1-235908574-235909512 | chr1-235866574-235867460 | 0.480820778 |
| chr1-235908574-235909512 | chr1-235883158-235884045 | 0.512758602 |
| chr1-235908574-235909512 | chr1-235897881-235898748 | 0.353817951 |
| chr1-235908574-235909512 | chr1-235910249-235911178 | 0.344479994 |
| chr1-235908574-235909512 | chr1-235917044-235918065 | 0.280865736 |
| chr1-235908574-235909512 | chr1-235954587-235955477 | 0.267544538 |
| chr1-235908574-235909512 | chr1-235971512-235972368 | 0.584786414 |
| chr1-235908574-235909512 | chr1-235999113-236000019 | 0.347718843 |
| chr1-235908574-235909512 | chr1-236018777-236019695 | 0.48267935 |
| chr1-235908574-235909512 | chr1-236039458-236040362 | 0.269287135 |
| chr1-235908574-235909512 | chr1-236063677-236064461 | 0.431988034 |
| chr1-235908574-235909512 | chr1-236064778-236065665 | 0.457642554 |
| chr1-235908574-235909512 | chr1-236142002-236142851 | 0.408905121 |
| chrX-1454159-1454795 | chrX-1657431-1658373 | 0.30626021 |
| chr11-11218451-11219294 | chr11-10792198-10793103 | 0.35582183 |
| chr11-11218451-11219294 | chr11-11147907-11148782 | 0.453466744 |
| chr11-11218451-11219294 | chr11-11396046-11396951 | 0.566289311 |
| chr1-67776889-67777871 | chr1-67535143-67535971 | 0.421415226 |
| chr1-67776889-67777871 | chr1-67558922-67559865 | 0.407731086 |
| chr1-67776889-67777871 | chr1-67563980-67564904 | 0.26759005 |
| chr1-67776889-67777871 | chr1-67583043-67583908 | 0.359546597 |
| chr1-67776889-67777871 | chr1-67586382-67587249 | 0.27888114 |
| chr1-67776889-67777871 | chr1-67612538-67613465 | 0.266772109 |
| chr1-67776889-67777871 | chr1-67618320-67619260 | 0.32709908 |
| chr1-67776889-67777871 | chr1-67645493-67646238 | 0.282858931 |
| chr1-67776889-67777871 | chr1-67652647-67653561 | 0.261312322 |
| chr1-67776889-67777871 | chr1-67684753-67685653 | 0.256116095 |
| chr1-67776889-67777871 | chr1-67686246-67687137 | 0.391801667 |
| chr1-67776889-67777871 | chr1-67710155-67711030 | 0.291888139 |
| chr1-67776889-67777871 | chr1-67746060-67746973 | 0.405232902 |
| chr1-67776889-67777871 | chr1-67759790-67760720 | 0.496875902 |
| chr1-67776889-67777871 | chr1-67815201-67816081 | 0.365797598 |
| chr1-67776889-67777871 | chr1-67831609-67832366 | 0.254251077 |
| chr1-67776889-67777871 | chr1-67832961-67833842 | 0.558876943 |
| chr1-67776889-67777871 | chr1-67987005-67987947 | 0.330397267 |
| chr1-67776889-67777871 | chr1-68173842-68174750 | 0.290996228 |
| chr1-67776889-67777871 | chr1-68214662-68215548 | 0.309434376 |
| chr1-202439164-202440029 | chr1-202438258-202439007 | 0.25846087 |
| chr1-42936096-42936989 | chr1-42681890-42682723 | 0.278472053 |
| chr1-204994895-204995782 | chr1-204633385-204634272 | 0.252098935 |
| chr1-204994895-204995782 | chr1-204828186-204829096 | 0.420228382 |
| chr1-204994895-204995782 | chr1-204829590-204830453 | 0.264552314 |
| chr1-204994895-204995782 | chr1-204887793-204888866 | 0.56329826 |
| chr1-204994895-204995782 | chr1-204980551-204981475 | 0.349369949 |
| chr1-204994895-204995782 | chr1-204989145-204990020 | 0.473932247 |
| chr1-204994895-204995782 | chr1-205061284-205062062 | 0.428050886 |
| chr1-204994895-204995782 | chr1-205121715-205122636 | 0.668770368 |
| chr1-204994895-204995782 | chr1-205175216-205176122 | 0.518103183 |
| chr1-204994895-204995782 | chr1-205199083-205199755 | 0.449388823 |
| chr1-204994895-204995782 | chr1-205211191-205212051 | 0.30430462 |
| chr1-204994895-204995782 | chr1-205227466-205228357 | 0.392454236 |
| chr11-14891795-14892508 | chr11-14904667-14905509 | 0.429357348 |
| chr11-14891795-14892508 | chr11-15034616-15035485 | 0.39522381 |
| chr1-183575542-183576288 | chr1-183281333-183282217 | 0.315791588 |
| chr1-183575542-183576288 | chr1-183471795-183472576 | 0.327954722 |
| chr1-183575542-183576288 | chr1-183576321-183577025 | 0.698585255 |
| chr1-183575542-183576288 | chr1-183635435-183636355 | 0.380676586 |
| chr1-183575542-183576288 | chr1-183942618-183943555 | 0.293581023 |
| chr1-183575542-183576288 | chr1-183958778-183959565 | 0.289267581 |
| chr19-46739059-46740179 | chr19-46346639-46347533 | 0.306671245 |
| chr19-46739059-46740179 | chr19-46413171-46414037 | 0.580951468 |
| chr19-46739059-46740179 | chr19-46471012-46471923 | 0.618952785 |
| chr19-46739059-46740179 | chr19-46600609-46601501 | -0.298184619 |
| chr19-46739059-46740179 | chr19-46624937-46625833 | 0.303156033 |
| chr19-46739059-46740179 | chr19-46829646-46830539 | 0.575510099 |
| chr19-46739059-46740179 | chr19-46840172-46841154 | 0.889570723 |
| chr19-46739059-46740179 | chr19-46855937-46856819 | 0.344613882 |
| chr1-230104445-230105325 | chr1-230084270-230085119 | 0.582014717 |
| chrX-150155251-150156200 | chrX-150163078-150164015 | 0.303154817 |
| chrX-2351792-2352708 | chrX-2461719-2462582 | 0.812883804 |
| chrX-2351792-2352708 | chrX-2593065-2593964 | 0.653513313 |
| chrX-2351792-2352708 | chrX-2693823-2694713 | 0.252141235 |
| chrX-136251002-136251805 | chrX-136055461-136056344 | 0.291433665 |
| chr1-159005367-159006283 | chr1-158999419-159000269 | 0.316808495 |
| chr1-159005367-159006283 | chr1-159009452-159010352 | 0.569666902 |
| chr1-58773204-58774064 | chr1-58546163-58547096 | 0.388191326 |
| chr1-58773204-58774064 | chr1-58584016-58584918 | 0.516354037 |
| chr1-58773204-58774064 | chr1-58618047-58618917 | 0.28367489 |
| chr1-58773204-58774064 | chr1-58718013-58719143 | 0.405772624 |
| chr1-58773204-58774064 | chr1-58727154-58728062 | 0.343427271 |
| chr1-58773204-58774064 | chr1-58783746-58784649 | 0.76100191 |
| chr1-58773204-58774064 | chr1-58785028-58785766 | 0.432567573 |
| chr1-58773204-58774064 | chr1-58815014-58815609 | 0.332502073 |
| chr1-58773204-58774064 | chr1-58848694-58849614 | 0.289157129 |
| chr1-58773204-58774064 | chr1-58874441-58875351 | 0.424549461 |
| chr1-58773204-58774064 | chr1-58885986-58886481 | 0.265175319 |
| chr1-58773204-58774064 | chr1-58903429-58904344 | 0.374485278 |
| chr1-58773204-58774064 | chr1-59007719-59008539 | 0.450952684 |
| chr1-58773204-58774064 | chr1-59013254-59014199 | 0.284273276 |
| chr1-58773204-58774064 | chr1-59019961-59020876 | 0.265877671 |
| chr1-58773204-58774064 | chr1-59025643-59026540 | 0.437981606 |
| chr1-58773204-58774064 | chr1-59056965-59057875 | 0.365972456 |
| chr1-58773204-58774064 | chr1-59156556-59157346 | 0.472374247 |
| chr1-67030888-67031847 | chr1-66751941-66752871 | 0.324888951 |
| chr1-67030888-67031847 | chr1-66924664-66925614 | 0.541071436 |
| chr1-67030888-67031847 | chr1-66929829-66930704 | 0.514721962 |
| chr1-67030888-67031847 | chr1-66944423-66945264 | 0.288688306 |
| chr1-67030888-67031847 | chr1-66995789-66996716 | 0.407868565 |
| chr1-67030888-67031847 | chr1-67019443-67020337 | 0.413181158 |
| chr1-67030888-67031847 | chr1-67053430-67054314 | 0.341678028 |
| chr1-67030888-67031847 | chr1-67347473-67348465 | 0.267868707 |
| chr1-67030888-67031847 | chr1-67430138-67430977 | 0.346600353 |
| chrX-107700065-107700979 | chrX-107449191-107450055 | 0.646462208 |
| chrX-107700065-107700979 | chrX-107676636-107677493 | 0.415121949 |
| chrX-107700065-107700979 | chrX-107766451-107767384 | 0.4046298 |
| chrX-107700065-107700979 | chrX-107787520-107788382 | 0.362064924 |
| chr1-54227015-54227836 | chr1-53889433-53890325 | 0.252910368 |
| chr1-54227015-54227836 | chr1-53945551-53946434 | 0.51348579 |
| chr1-54227015-54227836 | chr1-53982103-53983019 | 0.331767932 |
| chr1-54227015-54227836 | chr1-54052970-54053888 | 0.493452052 |
| chr1-54227015-54227836 | chr1-54137347-54138424 | 0.311571159 |
| chr1-54227015-54227836 | chr1-54153480-54154330 | 0.381642626 |
| chr1-54227015-54227836 | chr1-54199656-54200567 | 0.550624788 |
| chr1-54227015-54227836 | chr1-54220003-54220868 | 0.633562768 |
| chr1-54227015-54227836 | chr1-54249741-54250655 | 0.369942674 |
| chr1-54227015-54227836 | chr1-54253385-54254121 | 0.468590021 |
| chr1-54227015-54227836 | chr1-54390531-54391341 | 0.321432097 |
| chr1-54227015-54227836 | chr1-54406005-54406922 | 0.464159738 |
| chr1-54227015-54227836 | chr1-54407429-54408110 | 0.33213318 |
| chr1-54227015-54227836 | chr1-54487925-54488767 | 0.399889017 |
| chr1-54227015-54227836 | chr1-54489149-54489969 | 0.378829142 |
| chr19-1051922-1052830 | chr19-789025-789856 | 0.336872634 |
| chr19-1051922-1052830 | chr19-796882-797622 | 0.440145309 |
| chr19-1051922-1052830 | chr19-797743-798096 | 0.457867272 |
| chr19-1051922-1052830 | chr19-892800-893732 | 0.284415024 |
| chr19-1051922-1052830 | chr19-921317-922295 | 0.304436842 |
| chr19-1051922-1052830 | chr19-1020931-1021779 | 0.311627389 |
| chr19-1051922-1052830 | chr19-1039549-1040435 | 0.272690033 |
| chr19-1051922-1052830 | chr19-1066659-1067558 | 0.282673103 |
| chr19-1051922-1052830 | chr19-1069040-1069914 | 0.26652712 |
| chr19-1051922-1052830 | chr19-1094995-1095912 | 0.424035634 |
| chr19-1051922-1052830 | chr19-1103602-1104372 | 0.396587354 |
| chr19-1051922-1052830 | chr19-1150733-1151506 | 0.322465299 |
| chr19-1051922-1052830 | chr19-1154733-1155644 | 0.274148976 |
| chr19-1051922-1052830 | chr19-1205193-1206112 | 0.301483057 |
| chr19-1051922-1052830 | chr19-1221218-1222156 | 0.329587225 |
| chr19-1051922-1052830 | chr19-1237574-1238373 | 0.28320407 |
| chr19-1051922-1052830 | chr19-1239877-1240722 | 0.263812499 |
| chr19-1051922-1052830 | chr19-1241361-1242202 | 0.374702253 |
| chr19-1051922-1052830 | chr19-1248192-1249065 | 0.44122875 |
| chr19-1051922-1052830 | chr19-1249239-1250019 | 0.480785334 |
| chr19-1051922-1052830 | chr19-1252958-1253400 | 0.267746913 |
| chr19-1051922-1052830 | chr19-1266363-1267199 | 0.410772485 |
| chr19-1051922-1052830 | chr19-1268941-1269749 | 0.389035527 |
| chr19-1051922-1052830 | chr19-1275206-1276113 | 0.282377726 |
| chr19-1051922-1052830 | chr19-1354451-1355371 | 0.35870698 |
| chr19-1051922-1052830 | chr19-1383212-1384091 | 0.436244256 |
| chr19-1051922-1052830 | chr19-1406992-1407901 | 0.267163931 |
| chr19-1051922-1052830 | chr19-1437956-1438820 | 0.318365174 |
| chr19-1051922-1052830 | chr19-1444846-1445717 | 0.338545764 |
| chr19-1051922-1052830 | chr19-1490455-1491308 | 0.455377167 |
| chr11-61460240-61461312 | chr11-61041757-61042692 | 0.361467602 |
| chr11-61460240-61461312 | chr11-61681988-61682876 | 0.525039772 |
| chr11-61460240-61461312 | chr11-61695679-61696476 | 0.555680925 |
| chr11-61460240-61461312 | chr11-61747459-61748337 | 0.41982636 |
| chr10-30348807-30349721 | chr10-30182269-30183176 | 0.367866887 |
| chr10-30348807-30349721 | chr10-30219889-30220766 | 0.257765294 |
| chr1-158010431-158011302 | chr1-158177459-158178344 | 0.575687999 |
| chr1-85849977-85850819 | chr1-85576478-85577344 | 0.335841169 |
| chr1-85849977-85850819 | chr1-85707966-85708885 | 0.293695482 |
| chr1-85849977-85850819 | chr1-85798788-85799615 | 0.312685612 |
| chrX-107721233-107722021 | chrX-107484836-107485373 | 0.39762298 |
| chrX-107721233-107722021 | chrX-107673911-107674533 | 0.268211536 |
| chrX-107721233-107722021 | chrX-107712327-107713215 | 0.730028555 |
| chrX-107721233-107722021 | chrX-107716581-107717393 | 0.322836621 |
| chr10-80467722-80468626 | chr10-80464565-80465447 | 0.517891458 |
| chr10-80467722-80468626 | chr10-80497280-80498082 | 0.389774861 |
| chr1-8076850-8077716 | chr1-7770859-7771744 | 0.280192223 |
| chr1-8076850-8077716 | chr1-7783611-7784508 | 0.317559002 |
| chr1-8076850-8077716 | chr1-8025884-8026802 | 0.487096991 |
| chr1-8076850-8077716 | chr1-8040332-8041245 | 0.373354318 |
| chr1-8076850-8077716 | chr1-8047636-8048573 | 0.430369251 |
| chr1-8076850-8077716 | chr1-8090001-8090875 | 0.258065572 |
| chr1-8076850-8077716 | chr1-8094421-8095349 | 0.347287681 |
| chr1-8076850-8077716 | chr1-8116772-8117634 | 0.51687695 |
| chr1-8076850-8077716 | chr1-8121050-8121940 | 0.321447341 |
| chr1-8076850-8077716 | chr1-8133521-8134404 | 0.282900097 |
| chr1-8076850-8077716 | chr1-8169473-8170369 | 0.266237029 |
| chr1-8076850-8077716 | chr1-8197515-8198383 | 0.41634777 |
| chr1-8076850-8077716 | chr1-8211584-8212388 | 0.414894621 |
| chr1-8076850-8077716 | chr1-8283907-8284785 | 0.279389896 |
| chr1-8076850-8077716 | chr1-8294039-8294978 | 0.250823485 |
| chr1-8076850-8077716 | chr1-8343139-8344026 | 0.326981773 |
| chr1-8076850-8077716 | chr1-8348818-8349631 | 0.390355844 |
| chr1-8076850-8077716 | chr1-8396856-8397761 | 0.304203702 |
| chr1-8076850-8077716 | chr1-8423504-8424353 | 0.272379942 |
| chr10-75894476-75895474 | chr10-75662484-75663399 | 0.385080615 |
| chr1-234326350-234327319 | chr1-234102397-234103217 | 0.343701138 |
| chr1-234326350-234327319 | chr1-234352154-234353152 | 0.305205597 |
| chr1-234326350-234327319 | chr1-234478326-234479275 | 0.562031985 |
| chr1-234326350-234327319 | chr1-234498932-234499753 | 0.420146351 |
| chr1-234326350-234327319 | chr1-234503176-234504142 | 0.408094059 |
| chr1-234326350-234327319 | chr1-234515455-234516319 | 0.382863867 |
| chr1-234326350-234327319 | chr1-234523323-234524209 | 0.309247465 |
| chr1-234326350-234327319 | chr1-234531407-234532213 | 0.285690133 |
| chr1-234326350-234327319 | chr1-234544049-234544920 | 0.409110084 |
| chr1-234326350-234327319 | chr1-234582483-234583311 | 0.343902894 |
| chr1-234326350-234327319 | chr1-234585130-234586024 | 0.371892703 |
| chr1-234326350-234327319 | chr1-234599676-234600574 | 0.530161308 |
| chr1-234326350-234327319 | chr1-234609740-234610489 | 0.67865768 |
| chr1-234326350-234327319 | chr1-234610630-234611496 | 0.654219002 |
| chr1-234326350-234327319 | chr1-234656913-234657676 | 0.347199949 |
| chr1-234326350-234327319 | chr1-234683882-234684699 | 0.251069351 |
| chr1-234326350-234327319 | chr1-234699861-234700746 | 0.291531656 |
| chr1-234326350-234327319 | chr1-234702014-234702875 | 0.406532329 |
| chr1-234326350-234327319 | chr1-234717661-234718600 | 0.325366611 |
| chr1-234326350-234327319 | chr1-234721380-234722298 | 0.2633574 |
| chr1-234326350-234327319 | chr1-234723965-234724857 | 0.659797077 |
| chr11-11036553-11037396 | chr11-11391592-11392492 | 0.380563637 |
| chr1-40374391-40375140 | chr1-40040178-40040959 | 0.342397551 |
| chr1-40374391-40375140 | chr1-40076647-40077545 | 0.291974279 |
| chr1-40374391-40375140 | chr1-40084450-40085451 | 0.262344039 |
| chr1-40374391-40375140 | chr1-40160939-40161799 | 0.562932182 |
| chr1-40374391-40375140 | chr1-40257576-40258488 | 0.353806577 |
| chr1-40374391-40375140 | chr1-40315032-40315921 | 0.427436745 |
| chr1-40374391-40375140 | chr1-40373180-40374081 | 0.482470443 |
| chr1-40374391-40375140 | chr1-40385732-40386603 | 0.258343795 |
| chr1-40374391-40375140 | chr1-40508310-40509202 | 0.272450289 |
| chr1-40374391-40375140 | chr1-40612084-40612962 | 0.38887322 |
| chr1-40374391-40375140 | chr1-40691421-40692191 | 0.394326804 |
| chr1-88497745-88498487 | chr1-88180268-88181132 | 0.389889183 |
| chr11-19723318-19724216 | chr11-19890010-19890901 | 0.372340115 |
| chr10-99731805-99732690 | chr10-99845575-99846476 | -0.349863762 |
| chr1-45550358-45551283 | chr1-45265992-45266885 | 0.333528547 |
| chr1-45550358-45551283 | chr1-45299931-45300842 | 0.261647498 |
| chr1-45550358-45551283 | chr1-45303347-45304226 | 0.651872862 |
| chr1-45550358-45551283 | chr1-45326521-45327286 | 0.505249961 |
| chr1-45550358-45551283 | chr1-45490791-45491670 | 0.451228679 |
| chr1-45550358-45551283 | chr1-45499712-45500621 | 0.347088046 |
| chr1-45550358-45551283 | chr1-45521500-45522418 | 0.447426176 |
| chr1-45550358-45551283 | chr1-45583225-45584047 | 0.746929677 |
| chr1-45550358-45551283 | chr1-45584434-45585259 | 0.70150742 |
| chr1-45550358-45551283 | chr1-45686411-45686858 | 0.482895696 |
| chr1-45550358-45551283 | chr1-45750228-45751142 | 0.536405416 |
| chr1-45550358-45551283 | chr1-45802783-45803109 | 0.331502965 |
| chr1-45550358-45551283 | chr1-45803210-45803817 | 0.471533618 |
| chr1-45550358-45551283 | chr1-45954078-45954839 | 0.34018598 |
| chrX-107484836-107485373 | chrX-107712327-107713215 | 0.434471181 |
| chrX-107484836-107485373 | chrX-107721233-107722021 | 0.39762298 |
| chrX-20374273-20374818 | chrX-20374939-20375662 | 0.677468888 |
| chrX-110449795-110450687 | chrX-110146348-110147128 | 0.259275903 |
| chrX-110449795-110450687 | chrX-110244734-110245639 | 0.670051215 |
| chr1-1723971-1724877 | chr1-1273490-1274416 | 0.48009192 |
| chr1-1723971-1724877 | chr1-1304371-1305142 | 0.422184412 |
| chr1-1723971-1724877 | chr1-1307879-1308731 | 0.415435526 |
| chr1-1723971-1724877 | chr1-1317999-1318831 | 0.292571638 |
| chr1-1723971-1724877 | chr1-1324303-1325190 | 0.472951925 |
| chr1-1723971-1724877 | chr1-1349062-1350000 | 0.417882512 |
| chr1-1723971-1724877 | chr1-1358244-1359127 | 0.335872463 |
| chr1-1723971-1724877 | chr1-1371502-1372412 | 0.391008625 |
| chr1-1723971-1724877 | chr1-1374937-1375843 | 0.510325149 |
| chr1-1723971-1724877 | chr1-1399004-1399913 | 0.47953406 |
| chr1-1723971-1724877 | chr1-1406870-1407774 | 0.295070492 |
| chr1-1723971-1724877 | chr1-1419725-1420629 | 0.385197664 |
| chr1-1723971-1724877 | chr1-1427208-1428013 | 0.445994311 |
| chr1-1723971-1724877 | chr1-1430051-1430959 | 0.539875512 |
| chr1-1723971-1724877 | chr1-1433728-1434527 | 0.277841073 |
| chr1-1723971-1724877 | chr1-1511537-1512411 | 0.554547849 |
| chr1-1723971-1724877 | chr1-1540151-1541070 | 0.348312434 |
| chr1-1723971-1724877 | chr1-1574354-1575269 | 0.437427137 |
| chr1-1723971-1724877 | chr1-1615160-1615952 | 0.5985026 |
| chr1-1723971-1724877 | chr1-1658650-1659551 | 0.379069112 |
| chr1-1723971-1724877 | chr1-1692406-1693414 | 0.491007011 |
| chr1-1723971-1724877 | chr1-1745969-1746853 | 0.527723635 |
| chr1-1723971-1724877 | chr1-1747027-1747896 | 0.416813802 |
| chr1-1723971-1724877 | chr1-1748510-1749444 | 0.278121383 |
| chr1-1723971-1724877 | chr1-1777992-1778891 | 0.372082738 |
| chr1-1723971-1724877 | chr1-1782486-1783371 | 0.26000679 |
| chr1-1723971-1724877 | chr1-1858497-1859343 | 0.365030511 |
| chr1-1723971-1724877 | chr1-1890692-1891555 | 0.372230889 |
| chr1-1723971-1724877 | chr1-1905968-1906851 | 0.344359569 |
| chr1-1723971-1724877 | chr1-1908749-1909646 | 0.535065326 |
| chr1-1723971-1724877 | chr1-1919001-1919917 | 0.49905636 |
| chr1-1723971-1724877 | chr1-1943696-1944414 | 0.270299443 |
| chr1-1723971-1724877 | chr1-1959652-1960775 | 0.37717414 |
| chr1-1723971-1724877 | chr1-1989891-1990926 | 0.289560829 |
| chr19-37293128-37293958 | chr19-37294090-37294841 | 0.369543267 |
| chr1-8817341-8818256 | chr1-8517573-8518497 | 0.323997867 |
| chr1-8817341-8818256 | chr1-8686689-8687606 | 0.298516201 |
| chr1-8817341-8818256 | chr1-8727709-8728550 | 0.252619087 |
| chr1-8817341-8818256 | chr1-8825718-8826637 | 0.378526282 |
| chr1-8817341-8818256 | chr1-8848596-8849483 | 0.43922449 |
| chr1-8817341-8818256 | chr1-8873290-8874182 | 0.275263006 |
| chr1-8817341-8818256 | chr1-8878418-8879314 | 0.335718204 |
| chr1-8817341-8818256 | chr1-8880046-8880803 | 0.429179692 |
| chr1-8817341-8818256 | chr1-8889805-8890634 | 0.61255456 |
| chr1-8817341-8818256 | chr1-8917427-8918632 | 0.357099997 |
| chr1-8817341-8818256 | chr1-8985900-8986823 | 0.290808061 |
| chr1-8817341-8818256 | chr1-8987835-8988624 | 0.378652146 |
| chr1-8817341-8818256 | chr1-9004848-9005743 | 0.534229759 |
| chr1-8817341-8818256 | chr1-9061373-9062346 | 0.257265528 |
| chr1-8817341-8818256 | chr1-9066868-9067790 | 0.329980079 |
| chr1-8817341-8818256 | chr1-9069351-9070213 | 0.368622179 |
| chr1-8817341-8818256 | chr1-9110572-9111443 | 0.280233149 |
| chr1-8817341-8818256 | chr1-9128801-9129710 | 0.59974204 |
| chr1-8817341-8818256 | chr1-9153990-9154947 | 0.466339053 |
| chr1-8817341-8818256 | chr1-9163556-9164477 | 0.571146825 |
| chr1-8817341-8818256 | chr1-9166090-9167038 | 0.420218914 |
| chr1-8817341-8818256 | chr1-9196114-9197023 | 0.292632873 |
| chr1-8817341-8818256 | chr1-9201287-9202121 | 0.469001278 |
| chr1-8817341-8818256 | chr1-9233006-9233755 | 0.644844156 |
| chr1-8817341-8818256 | chr1-9234385-9235233 | 0.478026098 |
| chr1-8817341-8818256 | chr1-9239500-9240412 | 0.471856742 |
| chr1-7744529-7745495 | chr1-7783611-7784508 | 0.255084204 |
| chr1-51660803-51661706 | chr1-51518906-51519740 | 0.359348007 |
| chr1-51660803-51661706 | chr1-51616786-51617626 | 0.410702951 |
| chr11-121479674-121480488 | chr11-121451739-121452667 | 0.337800227 |
| chr11-121479674-121480488 | chr11-121482300-121483160 | 0.454286027 |
| chr1-220776484-220777316 | chr1-220732791-220733681 | 0.332092828 |
| chr1-220776484-220777316 | chr1-220747902-220748799 | 0.272339131 |
| chr1-220776484-220777316 | chr1-220843331-220844213 | 0.362136064 |
| chr1-234373092-234374003 | chr1-234503176-234504142 | 0.401886337 |
| chr1-234373092-234374003 | chr1-234515455-234516319 | 0.343999266 |
| chr1-234373092-234374003 | chr1-234544049-234544920 | 0.409083927 |
| chr1-234373092-234374003 | chr1-234582483-234583311 | 0.361665879 |
| chr1-234373092-234374003 | chr1-234585130-234586024 | 0.335882127 |
| chr1-234373092-234374003 | chr1-234599676-234600574 | 0.594821476 |
| chr1-234373092-234374003 | chr1-234609740-234610489 | 0.407510288 |
| chr1-234373092-234374003 | chr1-234610630-234611496 | 0.341435205 |
| chr1-234373092-234374003 | chr1-234702014-234702875 | 0.275153954 |
| chr1-234373092-234374003 | chr1-234717661-234718600 | 0.306170286 |
| chr1-234373092-234374003 | chr1-234721380-234722298 | 0.259320856 |
| chr1-234373092-234374003 | chr1-234723965-234724857 | 0.357292838 |
| chr1-112025706-112026528 | chr1-111755253-111756173 | 0.493147751 |
| chr1-112025706-112026528 | chr1-112360043-112360908 | 0.371005573 |
| chr1-112025706-112026528 | chr1-112393824-112394629 | 0.351696555 |
| chr19-19121084-19122027 | chr19-18784901-18785831 | 0.250337047 |
| chr19-19121084-19122027 | chr19-18789681-18790534 | 0.283480264 |
| chr19-19121084-19122027 | chr19-18793275-18794138 | 0.284443512 |
| chr19-19121084-19122027 | chr19-18986540-18987384 | 0.416451397 |
| chr19-19121084-19122027 | chr19-19033178-19034091 | 0.348984082 |
| chr19-19121084-19122027 | chr19-19134580-19135674 | 0.449118753 |
| chr19-19121084-19122027 | chr19-19394538-19395419 | 0.30013316 |
| chr10-74081040-74081683 | chr10-74081799-74082564 | 0.385609989 |
| chr10-5977197-5978107 | chr10-5545678-5546577 | 0.26014502 |
| chr10-5977197-5978107 | chr10-5684268-5685148 | 0.267099128 |
| chr10-5977197-5978107 | chr10-5852127-5853080 | 0.340107323 |
| chr10-5977197-5978107 | chr10-6144402-6145310 | 0.25274806 |
| chr1-209723565-209724463 | chr1-209692551-209693391 | 0.276284101 |
| chr1-209723565-209724463 | chr1-209769124-209770012 | 0.299262623 |
| chr1-205321294-205322137 | chr1-205141396-205142270 | 0.302394881 |
| chr1-205321294-205322137 | chr1-205528686-205529608 | 0.293338022 |
| chr1-205321294-205322137 | chr1-205749065-205749903 | 0.277052502 |
| chr1-212234361-212235237 | chr1-211830222-211831058 | 0.424567992 |
| chr10-14779119-14779995 | chr10-14536353-14537305 | 0.553540851 |
| chr10-14779119-14779995 | chr10-14653550-14654414 | 0.251892295 |
| chr10-14779119-14779995 | chr10-14878269-14879138 | 0.417211758 |
| chrX-137052146-137052981 | chrX-136880334-136881242 | 0.305885138 |
| chr1-63615443-63616619 | chr1-63322981-63323712 | 0.310468278 |
| chr1-63615443-63616619 | chr1-63327481-63328393 | 0.291041376 |
| chr1-63615443-63616619 | chr1-63522925-63523835 | 0.32970237 |
| chr1-63615443-63616619 | chr1-63773627-63774420 | 0.344191748 |
| chr1-63615443-63616619 | chr1-63826394-63827099 | 0.322389277 |
| chr1-63615443-63616619 | chr1-63860832-63861704 | 0.281772418 |
| chr10-3595512-3596045 | chr10-3424124-3424965 | 0.362446817 |
| chr11-7629648-7630657 | chr11-7251590-7252363 | 0.502622827 |
| chr11-7629648-7630657 | chr11-7512358-7513169 | 0.280131521 |
| chr11-7629648-7630657 | chr11-7513355-7514242 | 0.497930493 |
| chr11-7629648-7630657 | chr11-7688020-7689030 | 0.330745935 |
| chr1-204941440-204942339 | chr1-204515945-204516864 | 0.304521766 |
| chr1-204941440-204942339 | chr1-204980551-204981475 | 0.350230584 |
| chr1-204941440-204942339 | chr1-205211191-205212051 | 0.283419506 |
| chr1-204941440-204942339 | chr1-205227466-205228357 | 0.294619842 |
| chr1-155017050-155017981 | chr1-154869951-154870824 | 0.326953824 |
| chr1-155017050-155017981 | chr1-154936636-154937581 | 0.522770917 |
| chr1-155017050-155017981 | chr1-154940937-154941845 | 0.348484953 |
| chr1-155017050-155017981 | chr1-154955764-154956646 | 0.291296692 |
| chr1-155017050-155017981 | chr1-154961327-154962210 | 0.368407978 |
| chr1-155017050-155017981 | chr1-154970521-154971411 | 0.486136433 |
| chr1-155017050-155017981 | chr1-154972334-154973226 | 0.456959273 |
| chr1-155017050-155017981 | chr1-154974118-154975022 | 0.538171211 |
| chr1-155017050-155017981 | chr1-154982776-154983684 | 0.285503098 |
| chr1-155017050-155017981 | chr1-155000497-155001313 | 0.453858692 |
| chr1-155017050-155017981 | chr1-155001792-155002672 | 0.458342436 |
| chr1-155017050-155017981 | chr1-155050823-155051508 | 0.403282252 |
| chr1-155017050-155017981 | chr1-155061231-155062093 | 0.520417715 |
| chr1-155017050-155017981 | chr1-155063370-155064228 | 0.409195802 |
| chr1-155017050-155017981 | chr1-155078398-155079267 | 0.270025633 |
| chr1-155017050-155017981 | chr1-155084664-155085569 | 0.467606338 |
| chr1-155017050-155017981 | chr1-155091126-155091986 | 0.399773376 |
| chr1-155017050-155017981 | chr1-155135337-155136249 | 0.477127363 |
| chr1-155017050-155017981 | chr1-155166218-155167141 | 0.40853338 |
| chr1-155017050-155017981 | chr1-155167516-155168391 | 0.313725593 |
| chr1-155017050-155017981 | chr1-155172779-155173644 | 0.502599594 |
| chr1-155017050-155017981 | chr1-155176377-155177274 | 0.353343609 |
| chr1-155017050-155017981 | chr1-155193112-155193970 | 0.394684823 |
| chr1-155017050-155017981 | chr1-155194537-155195367 | 0.339304107 |
| chr1-155017050-155017981 | chr1-155206651-155207420 | 0.424033179 |
| chr1-155017050-155017981 | chr1-155208413-155209286 | 0.395868009 |
| chr1-155017050-155017981 | chr1-155211410-155212329 | 0.379935075 |
| chr1-155017050-155017981 | chr1-155227185-155228083 | 0.497176248 |
| chr1-155017050-155017981 | chr1-155250401-155251304 | 0.363793635 |
| chr1-155017050-155017981 | chr1-155261901-155262771 | 0.618304041 |
| chr1-155017050-155017981 | chr1-155273064-155273946 | 0.509629135 |
| chr1-155017050-155017981 | chr1-155276811-155277811 | 0.439730097 |
| chr1-155017050-155017981 | chr1-155308272-155309187 | 0.478677762 |
| chr1-155017050-155017981 | chr1-155320613-155321333 | 0.407014687 |
| chr1-155017050-155017981 | chr1-155323779-155324721 | 0.396134565 |
| chr1-155017050-155017981 | chr1-155379340-155380215 | 0.361197775 |
| chr1-155017050-155017981 | chr1-155463563-155464516 | 0.315664816 |
| chr10-77537341-77538285 | chr10-77939497-77940424 | 0.262106274 |
| chr1-39173220-39174131 | chr1-39047380-39048274 | 0.439750867 |
| chr1-39173220-39174131 | chr1-39104888-39105784 | 0.319097464 |
| chr1-39173220-39174131 | chr1-39110303-39111252 | 0.252554105 |
| chr1-39173220-39174131 | chr1-39183486-39184532 | 0.474201652 |
| chr1-39173220-39174131 | chr1-39191834-39192729 | 0.438980273 |
| chr1-39173220-39174131 | chr1-39408403-39409298 | 0.319410075 |
| chr10-93496051-93496927 | chr10-93088009-93089012 | 0.290927129 |
| chr10-93496051-93496927 | chr10-93165116-93166025 | 0.484335909 |
| chr10-93496051-93496927 | chr10-93186415-93187283 | 0.442992827 |
| chr10-93496051-93496927 | chr10-93468232-93469120 | 0.733534835 |
| chr10-93496051-93496927 | chr10-93482069-93482944 | 0.573198837 |
| chr10-93496051-93496927 | chr10-93515097-93515983 | 0.413863491 |
| chr10-93496051-93496927 | chr10-93595615-93596464 | 0.527490183 |
| chr1-151763058-151763957 | chr1-151608028-151608936 | 0.481664559 |
| chr1-151763058-151763957 | chr1-151611591-151612491 | 0.311080659 |
| chr1-151763058-151763957 | chr1-151734808-151735688 | 0.355163496 |
| chr1-151763058-151763957 | chr1-151909138-151910033 | 0.347915621 |
| chr1-151763058-151763957 | chr1-151941078-151942005 | 0.35926305 |
| chr1-151763058-151763957 | chr1-151945493-151946375 | 0.402240311 |
| chr1-151763058-151763957 | chr1-151960872-151961774 | 0.391878409 |
| chr1-151763058-151763957 | chr1-151976516-151977431 | 0.270687194 |
| chr1-151763058-151763957 | chr1-151980146-151981019 | 0.534026202 |
| chr1-151763058-151763957 | chr1-151992216-151993045 | 0.353586049 |
| chr1-151763058-151763957 | chr1-151994811-151995684 | 0.270403801 |
| chr1-151763058-151763957 | chr1-152001369-152001999 | 0.542125153 |
| chr1-151763058-151763957 | chr1-152034248-152035149 | 0.642152455 |
| chr1-151763058-151763957 | chr1-152036584-152037399 | 0.596813564 |
| chr1-151763058-151763957 | chr1-152047717-152048633 | 0.287874587 |
| chr1-151763058-151763957 | chr1-152052396-152053217 | 0.628932788 |
| chr1-151763058-151763957 | chr1-152112246-152113235 | 0.260782879 |
| chr1-151763058-151763957 | chr1-152185255-152186151 | 0.377764458 |
| chr1-151763058-151763957 | chr1-152188578-152189427 | 0.468761106 |
| chr1-226870290-226871183 | chr1-226738975-226739867 | 0.277199888 |
| chr1-63898631-63899517 | chr1-63674205-63675101 | 0.26055104 |
| chr1-63898631-63899517 | chr1-63964972-63965849 | 0.304323454 |
| chr1-63898631-63899517 | chr1-64132866-64133713 | 0.273807507 |
| chr1-203769230-203770146 | chr1-203557345-203558026 | 0.438516407 |
| chr1-203769230-203770146 | chr1-203626330-203627249 | 0.454478363 |
| chr1-203769230-203770146 | chr1-203672000-203672870 | 0.448518888 |
| chr1-203769230-203770146 | chr1-203692622-203693531 | 0.300322557 |
| chr1-203769230-203770146 | chr1-203701745-203702670 | 0.371832825 |
| chr1-203769230-203770146 | chr1-203986743-203987728 | 0.405662379 |
| chr1-203769230-203770146 | chr1-204088770-204089677 | 0.259171172 |
| chr1-203769230-203770146 | chr1-204206817-204207721 | 0.334691026 |
| chr1-243803256-243804096 | chr1-244047927-244048835 | 0.522423462 |
| chr1-243803256-243804096 | chr1-244103878-244104996 | 0.286976139 |
| chr1-11663930-11664730 | chr1-11272722-11273610 | 0.290293864 |
| chr1-11663930-11664730 | chr1-11654424-11655224 | 0.377313136 |
| chr1-11663930-11664730 | chr1-11680781-11681699 | 0.425449736 |
| chr1-11663930-11664730 | chr1-11719359-11720165 | 0.255793327 |
| chr1-11663930-11664730 | chr1-11731556-11732389 | 0.255526728 |
| chr1-11663930-11664730 | chr1-11787309-11788147 | 0.397609569 |
| chr1-11663930-11664730 | chr1-11790599-11791508 | 0.347819596 |
| chr1-11663930-11664730 | chr1-11805564-11806482 | 0.545308113 |
| chr1-11663930-11664730 | chr1-11858988-11859822 | 0.259007709 |
| chr1-11663930-11664730 | chr1-11874076-11874882 | 0.366500837 |
| chr1-11663930-11664730 | chr1-11893686-11894582 | 0.356570193 |
| chr1-11663930-11664730 | chr1-11907659-11908553 | 0.421445185 |
| chr1-11663930-11664730 | chr1-11926035-11926944 | 0.757734006 |
| chr1-11663930-11664730 | chr1-11929541-11930427 | 0.39047932 |
| chr1-11663930-11664730 | chr1-11938799-11939661 | 0.368616946 |
| chr1-11663930-11664730 | chr1-11979763-11980659 | 0.53332868 |
| chr1-161749404-161750244 | chr1-161305738-161306637 | 0.433740823 |
| chr1-161749404-161750244 | chr1-161313877-161314795 | 0.412770822 |
| chr1-161749404-161750244 | chr1-161382111-161383041 | 0.465642422 |
| chr1-161749404-161750244 | chr1-161389656-161390565 | 0.618694399 |
| chr1-161749404-161750244 | chr1-161399344-161400156 | 0.425573746 |
| chr1-161749404-161750244 | chr1-161486932-161487778 | 0.3458661 |
| chr1-161749404-161750244 | chr1-161530598-161531394 | 0.364399143 |
| chr1-161749404-161750244 | chr1-161532581-161533484 | 0.384766372 |
| chr1-161749404-161750244 | chr1-161612132-161613056 | 0.271248819 |
| chr1-161749404-161750244 | chr1-161726645-161727533 | 0.321821206 |
| chr1-161749404-161750244 | chr1-161738506-161739410 | 0.288405133 |
| chr1-161749404-161750244 | chr1-161764822-161765626 | 0.408126306 |
| chr1-161749404-161750244 | chr1-161792217-161793157 | 0.351871581 |
| chr1-161749404-161750244 | chr1-161973912-161974769 | 0.367505409 |
| chr1-161749404-161750244 | chr1-161986068-161986965 | 0.298782404 |
| chr1-161749404-161750244 | chr1-162002955-162003782 | 0.291060048 |
| chr1-161749404-161750244 | chr1-162023519-162024342 | 0.356453409 |
| chr1-161749404-161750244 | chr1-162069333-162070188 | 0.39804536 |
| chr11-133980857-133981822 | chr11-133927367-133928297 | 0.359956788 |
| chr11-133980857-133981822 | chr11-133945187-133946155 | 0.558915003 |
| chr11-133980857-133981822 | chr11-134068634-134069483 | 0.277539619 |
| chr11-133980857-133981822 | chr11-134120005-134120738 | 0.269564044 |
| chr11-133980857-133981822 | chr11-134124753-134125620 | 0.767351396 |
| chr1-161532581-161533484 | chr1-161305738-161306637 | 0.590225812 |
| chr1-161532581-161533484 | chr1-161313877-161314795 | 0.479807953 |
| chr1-161532581-161533484 | chr1-161382111-161383041 | 0.522343902 |
| chr1-161532581-161533484 | chr1-161389656-161390565 | 0.64649833 |
| chr1-161532581-161533484 | chr1-161399344-161400156 | 0.53903186 |
| chr1-161532581-161533484 | chr1-161486932-161487778 | 0.450799802 |
| chr1-161532581-161533484 | chr1-161530598-161531394 | 0.445718316 |
| chr1-161532581-161533484 | chr1-161560955-161561874 | 0.477426514 |
| chr1-161532581-161533484 | chr1-161726645-161727533 | 0.413872543 |
| chr1-161532581-161533484 | chr1-161738506-161739410 | 0.64249964 |
| chr1-161532581-161533484 | chr1-161749404-161750244 | 0.384766372 |
| chr1-161532581-161533484 | chr1-161764822-161765626 | 0.546527313 |
| chr1-161532581-161533484 | chr1-161792217-161793157 | 0.259277921 |
| chr1-161532581-161533484 | chr1-161929836-161930720 | 0.566385353 |
| chr1-161532581-161533484 | chr1-161973912-161974769 | 0.313948339 |
| chr1-161532581-161533484 | chr1-161993989-161994886 | 0.323058055 |
| chr1-209576569-209577346 | chr1-209569150-209570059 | 0.732365894 |
| chr1-209576569-209577346 | chr1-209641334-209642321 | 0.41385244 |
| chr1-209576569-209577346 | chr1-209648911-209649861 | 0.318837744 |
| chr1-209576569-209577346 | chr1-209652826-209653724 | 0.431681702 |
| chr1-209576569-209577346 | chr1-209675027-209675892 | 0.282188066 |
| chr1-209576569-209577346 | chr1-209747458-209748365 | 0.409205461 |
| chr1-209576569-209577346 | chr1-209884462-209885333 | 0.370663569 |
| chr1-201028176-201029136 | chr1-200979308-200979989 | 0.264193538 |
| chr1-201028176-201029136 | chr1-201055719-201056591 | 0.320339489 |
| chr1-201028176-201029136 | chr1-201137440-201138305 | 0.533395184 |
| chr1-201028176-201029136 | chr1-201154010-201154914 | 0.549698011 |
| chr1-201028176-201029136 | chr1-201278895-201279831 | 0.315070477 |
| chr1-201028176-201029136 | chr1-201295678-201296651 | 0.407388122 |
| chr1-201028176-201029136 | chr1-201361381-201362247 | 0.424511128 |
| chr1-201028176-201029136 | chr1-201447958-201448812 | 0.605772688 |
| chr1-201028176-201029136 | chr1-201450623-201451532 | 0.584380594 |
| chr1-201028176-201029136 | chr1-201456582-201457443 | 0.427108488 |
| chr1-201028176-201029136 | chr1-201468817-201469672 | 0.456236972 |
| chr1-201028176-201029136 | chr1-201480196-201481127 | 0.29665487 |
| chr1-201028176-201029136 | chr1-201481735-201482613 | 0.597609006 |
| chr1-201028176-201029136 | chr1-201495536-201496453 | 0.275743476 |
| chr1-178650849-178651591 | chr1-178651711-178652226 | 0.505143231 |
| chr1-56394071-56395013 | chr1-56068083-56068865 | 0.56353464 |
| chr1-56394071-56395013 | chr1-56080971-56081851 | 0.324154274 |
| chr1-56394071-56395013 | chr1-56150301-56151255 | 0.289859232 |
| chr1-56394071-56395013 | chr1-56255472-56256340 | 0.569601175 |
| chr1-56394071-56395013 | chr1-56374011-56374889 | 0.374513559 |
| chr1-56394071-56395013 | chr1-56377506-56378125 | 0.563486675 |
| chr1-56394071-56395013 | chr1-56446442-56447483 | 0.488559394 |
| chr1-56394071-56395013 | chr1-56451950-56452830 | 0.446087765 |
| chr1-56394071-56395013 | chr1-56477203-56478131 | 0.383591951 |
| chr1-56394071-56395013 | chr1-56579098-56579956 | 0.257837653 |
| chr19-14253744-14254664 | chr19-14081031-14081950 | 0.252900115 |
| chr19-14253744-14254664 | chr19-14090575-14091491 | 0.44403098 |
| chr19-14253744-14254664 | chr19-14195769-14196644 | 0.357917438 |
| chr19-14253744-14254664 | chr19-14434706-14435635 | 0.466316081 |
| chr19-14253744-14254664 | chr19-14510506-14511375 | 0.64797766 |
| chr19-14253744-14254664 | chr19-14542516-14543424 | 0.430584101 |
| chr19-14253744-14254664 | chr19-14689432-14690345 | 0.251420932 |
| chrX-12970267-12971229 | chrX-12971373-12972240 | 0.283447756 |
| chr11-15818024-15818945 | chr11-15819470-15820322 | 0.372993289 |
| chrX-107712327-107713215 | chrX-107484836-107485373 | 0.434471181 |
| chrX-107712327-107713215 | chrX-107721233-107722021 | 0.730028555 |

**Supplementary Table 3.** The peak-to-gene linkages.

| gene | peak | p |
| --- | --- | --- |
| PRDX3 | chr10-42083190-42083984 | 1.26E-17 |
| PRDX5 | chr11-133980857-133981822 | 4.86E-16 |
| PRDX3 | chr10-114426424-114427269 | 1.42E-15 |
| PRDX4 | chrX-11469875-11470856 | 9.95E-13 |
| PRDX1 | chr1-84764199-84764665 | 2.72E-12 |
| PRDX1 | chr1-243803256-243804096 | 9.71E-12 |
| PRDX3 | chr10-74070845-74071724 | 1.08E-11 |
| PRDX6 | chr1-230104445-230105325 | 1.68E-11 |
| PRDX6 | chr1-204742243-204742966 | 2.68E-11 |
| PRDX4 | chrX-107721233-107722021 | 3.23E-10 |
| PRDX3 | chr10-3595512-3596045 | 6.70E-10 |
| PRDX6 | chr1-39173220-39174131 | 7.76E-10 |
| PRDX5 | chr11-61460240-61461312 | 1.35E-09 |
| PRDX3 | chr10-89207782-89208367 | 1.41E-09 |
| PRDX5 | chr11-68869517-68870387 | 1.70E-09 |
| PRDX6 | chr1-151763058-151763957 | 1.84E-09 |
| PRDX4 | chrX-67939297-67940152 | 1.98E-09 |
| PRDX4 | chrX-107484836-107485373 | 2.80E-09 |
| PRDX5 | chr11-11218451-11219294 | 4.27E-09 |
| PRDX5 | chr11-121479674-121480488 | 4.41E-09 |
| PRDX2 | chr19-37293128-37293958 | 5.44E-09 |
| PRDX6 | chr1-159005367-159006283 | 5.94E-09 |
| PRDX4 | chrX-150155251-150156200 | 1.19E-08 |
| PRDX5 | chr11-6910749-6911442 | 1.50E-08 |
| PRDX4 | chrX-129726164-129727109 | 1.60E-08 |
| PRDX1 | chr1-183575542-183576288 | 1.64E-08 |
| PRDX4 | chrX-20374273-20374818 | 1.68E-08 |
| PRDX5 | chr11-7629648-7630657 | 1.70E-08 |
| PRDX6 | chr1-234326350-234327319 | 1.90E-08 |
| PRDX6 | chr1-56394071-56395013 | 2.56E-08 |
| PRDX6 | chr1-45550358-45551283 | 3.16E-08 |
| PRDX6 | chr1-9397721-9398650 | 3.76E-08 |
| PRDX4 | chrX-110449795-110450687 | 3.90E-08 |
| PRDX2 | chr19-19121084-19122027 | 3.95E-08 |
| PRDX3 | chr10-132627895-132628666 | 4.17E-08 |
| PRDX1 | chr1-204994895-204995782 | 4.40E-08 |
| PRDX6 | chr1-11663930-11664730 | 5.10E-08 |
| PRDX6 | chr1-155017050-155017981 | 5.34E-08 |
| PRDX1 | chr1-161532581-161533484 | 6.33E-08 |
| PRDX3 | chr10-74168609-74169508 | 8.64E-08 |
| PRDX6 | chr1-213905101-213905959 | 1.20E-07 |
| PRDX4 | chrX-2577707-2578630 | 1.26E-07 |
| PRDX3 | chr10-99731805-99732690 | 1.28E-07 |
| PRDX5 | chr11-19723318-19724216 | 1.56E-07 |
| PRDX1 | chr1-220776484-220777316 | 1.60E-07 |
| PRDX4 | chrX-104921817-104922750 | 1.66E-07 |
| PRDX6 | chr1-118858510-118859318 | 1.77E-07 |
| PRDX1 | chr1-169311944-169312920 | 1.80E-07 |
| PRDX6 | chr1-193926957-193927730 | 1.81E-07 |
| PRDX5 | chr11-11370314-11371204 | 1.91E-07 |
| PRDX6 | chr1-203769230-203770146 | 2.06E-07 |
| PRDX4 | chrX-20394835-20395726 | 2.18E-07 |
| PRDX1 | chr1-63898631-63899517 | 2.23E-07 |
| PRDX3 | chr10-30348807-30349721 | 2.42E-07 |
| PRDX3 | chr10-78649806-78650616 | 2.43E-07 |
| PRDX6 | chr1-61435605-61436590 | 2.46E-07 |
| PRDX4 | chrX-19810192-19811279 | 2.54E-07 |
| PRDX6 | chr1-209576569-209577346 | 2.85E-07 |
| PRDX6 | chr1-235908574-235909512 | 3.07E-07 |
| PRDX4 | chrX-107700065-107700979 | 3.22E-07 |
| PRDX2 | chr19-14253744-14254664 | 3.53E-07 |
| PRDX6 | chr1-242243180-242244035 | 3.69E-07 |
| PRDX5 | chr11-11293512-11294570 | 4.59E-07 |
| PRDX3 | chr10-74081040-74081683 | 4.78E-07 |
| PRDX5 | chr11-60530477-60531317 | 5.01E-07 |
| PRDX3 | chr10-37495357-37496135 | 5.44E-07 |
| PRDX3 | chr10-93309643-93310056 | 5.67E-07 |
| PRDX2 | chr19-1051922-1052830 | 6.27E-07 |
| PRDX3 | chr10-93496051-93496927 | 7.14E-07 |
| PRDX6 | chr1-99657200-99657746 | 7.29E-07 |
| PRDX3 | chr10-126397555-126398657 | 8.03E-07 |
| PRDX5 | chr11-11036553-11037396 | 8.16E-07 |
| PRDX3 | chr10-12270586-12271469 | 8.28E-07 |
| PRDX6 | chr1-1723971-1724877 | 8.87E-07 |
| PRDX4 | chrX-1454159-1454795 | 9.12E-07 |
| PRDX6 | chr1-100459252-100460149 | 1.02E-06 |
| PRDX3 | chr10-75894476-75895474 | 1.15E-06 |
| PRDX1 | chr1-63615443-63616619 | 1.18E-06 |
| PRDX5 | chr11-14891795-14892508 | 1.23E-06 |
| PRDX4 | chrX-137052146-137052981 | 1.24E-06 |
| PRDX6 | chr1-161749404-161750244 | 1.24E-06 |
| PRDX3 | chr10-12531128-12532120 | 1.46E-06 |
| PRDX1 | chr1-158010431-158011302 | 1.48E-06 |
| PRDX1 | chr1-158818536-158819461 | 1.53E-06 |
| PRDX3 | chr10-77537341-77538285 | 1.53E-06 |
| PRDX6 | chr1-199852178-199852696 | 1.55E-06 |
| PRDX6 | chr1-202315340-202316152 | 1.64E-06 |
| PRDX1 | chr1-202439164-202440029 | 1.64E-06 |
| PRDX1 | chr1-68904219-68905118 | 1.70E-06 |
| PRDX6 | chr1-67776889-67777871 | 1.72E-06 |
| PRDX1 | chr1-85849977-85850819 | 1.80E-06 |
| PRDX5 | chr11-128848307-128849392 | 1.80E-06 |
| PRDX6 | chr1-204941440-204942339 | 1.82E-06 |
| PRDX6 | chr1-8817341-8818256 | 1.86E-06 |
| PRDX4 | chrX-115357887-115358811 | 2.02E-06 |
| PRDX5 | chr11-15818024-15818945 | 2.04E-06 |
| PRDX6 | chr1-7744529-7745495 | 2.05E-06 |
| PRDX6 | chr1-209723565-209724463 | 2.06E-06 |
| PRDX2 | chr19-46739059-46740179 | 2.08E-06 |
| PRDX6 | chr1-54227015-54227836 | 2.27E-06 |
| PRDX6 | chr1-40374391-40375140 | 2.28E-06 |
| PRDX4 | chrX-12970267-12971229 | 2.44E-06 |
| PRDX3 | chr10-78299697-78300601 | 2.44E-06 |
| PRDX6 | chr1-212234361-212235237 | 2.45E-06 |
| PRDX1 | chr1-67030888-67031847 | 2.46E-06 |
| PRDX4 | chrX-136251002-136251805 | 2.52E-06 |
| PRDX1 | chr1-178650849-178651591 | 2.85E-06 |
| PRDX6 | chr1-234373092-234374003 | 3.26E-06 |
| PRDX1 | chr1-206495431-206496282 | 3.31E-06 |
| PRDX3 | chr10-80467722-80468626 | 3.79E-06 |
| PRDX5 | chr11-119865230-119866306 | 3.83E-06 |
| PRDX6 | chr1-112025706-112026528 | 4.12E-06 |
| PRDX1 | chr1-158203605-158204681 | 4.35E-06 |
| PRDX4 | chrX-107712327-107713215 | 4.54E-06 |
| PRDX1 | chr1-77370669-77371531 | 4.61E-06 |
| PRDX1 | chr1-51660803-51661706 | 4.76E-06 |
| PRDX6 | chr1-205321294-205322137 | 4.84E-06 |
| PRDX6 | chr1-42936096-42936989 | 4.92E-06 |
| PRDX3 | chr10-14779119-14779995 | 5.34E-06 |
| PRDX4 | chrX-101415315-101416270 | 5.39E-06 |
| PRDX6 | chr1-27284517-27285345 | 5.45E-06 |
| PRDX6 | chr1-56424690-56425374 | 6.10E-06 |
| PRDX3 | chr10-5977197-5978107 | 6.30E-06 |
| PRDX4 | chrX-19554712-19555734 | 6.97E-06 |
| PRDX1 | chr1-14615536-14616011 | 7.12E-06 |
| PRDX6 | chr1-226870290-226871183 | 7.22E-06 |
| PRDX1 | chr1-88497745-88498487 | 7.25E-06 |
| PRDX4 | chrX-2351792-2352708 | 7.63E-06 |
| PRDX3 | chr10-126140637-126141630 | 7.73E-06 |
| PRDX6 | chr1-58773204-58774064 | 8.00E-06 |
| PRDX6 | chr1-8076850-8077716 | 8.37E-06 |
| PRDX6 | chr1-201028176-201029136 | 8.84E-06 |
